# Construction of a Genetic Linkage Map and Identification of QTLs for Seed Weight and Seed Size Traits in Lentil (*Lens culinaris* Medik.)

**DOI:** 10.1371/journal.pone.0139666

**Published:** 2015-10-05

**Authors:** Priyanka Verma, Richa Goyal, R. K. Chahota, Tilak R. Sharma, M. Z. Abdin, Sabhyata Bhatia

**Affiliations:** 1 National Institute of Plant Genome Research, Post Box No. 10531, Aruna Asaf Ali Marg, New Delhi, 110067, India; 2 Department of Agricultural Biotechnology, Chaudhary Sarwan Kumar Himachal Pradesh Agricultural University, Palampur, 176 062, India; 3 Department of Biotechnology, Faculty of Science, Jamia Hamdard, New Delhi, 110062, India; National Institute of Plant Genome Research (NIPGR), INDIA

## Abstract

Seed weight and seed size both are quantitative traits and have been considered as important components of grain yield, thus identification of quantitative trait loci (QTL) for seed traits in lentil (*Lens culinaris*) would be beneficial for the improvement of grain yield. Hence the main objective of this study was to identify QTLs for seed traits using an intraspecific mapping population derived from a cross between *L*. *culinaris* cv. Precoz (seed weight-5.1g, seed size-5.7mm) and *L*. *culinaris* cv. L830 (seed weight-2.2g, seed size-4mm) comprising 126 F_8_-RILs. For this, two microsatellite genomic libraries enriched for (GA/CT) and (GAA/CTT) motif were constructed which resulted in the development of 501 new genomic SSR markers. Six hundred forty seven SSR markers (including 146 previously published) were screened for parental polymorphism and 219 (33.8%) were found to be polymorphic among the parents. Of these 216 were mapped on seven linkage groups at LOD4.0 spanning 1183.7cM with an average marker density of 5.48cM. Phenotypic data from the RILs was used to identify QTLs for the seed weight and seed size traits by single marker analysis (SMA) followed by composite interval mapping (CIM) which resulted in one QTL each for the 2 traits (*qSW* and *qSS*) that were co-localized on LG4 and explained 48.4% and 27.5% of phenotypic variance respectively. The current study would serve as a strong foundation for further validation and fine mapping for utilization in lentil breeding programs.

## Introduction

Lentil (*Lens culinaris* Medik.) is an annual cool-season self-pollinated diploid (2x = 2n = 14) grain legume and is the world’s fifth largest pulse crop with an annual world production of 4.5 million metric tonnes with an average yield of 10,887 kg/ha (FAOSTAT, 2012). It’s production and consumption involves more than 100 countries (production: about 70 countries and consumption: >120 countries). It is an ancient crop that is believed to be originated in the Near East and later spread all through the Mediterranean Basin and central Asia [1]. The cultivated variety *L*. *culinaris* spp. *culinaris* encompasses two physio-morphological cultivated lentil types: small-seeded (microsperma) and large-seeded (macrosperma) [2]. The seeds are highly nutritious and contains almost all the essential elements for human consumption especially protein, carbohydrate, vitamins, micronutrients (K, P, Fe, Zn) and β-carotene [3]. Moreover, they are used as fodder for livestock, and generally grown in rotation to cereals to enrich the soil with their nitrogen fixing ability [4]. The genetic and genomic analysis of lentil is limited as compared to other legumes, due to the limited availability of molecular tools, the breeding programs in this legume crop leading to crop improvement is lacking. Thus in order to enable breeders to produce varieties with better yield and quality, efficient molecular tools like markers and dense linkage maps are required to boost the current crop improvement programs in this grain legume.

Molecular markers especially microsatellites are considered as an important tools for a number of genomic applications such as analysis of genetic diversity, construction of linkage map, mapping of qualitative and quantitative traits, map-based cloning of genes etc. [5]. They are hypervariable, co-dominant and are distributed ubiquitously throughout the genome [6]. Their high polymorphism rate which arises due to high mutation rate and random occurrence in the genome makes them more popular thus making them the most promising class of markers for construction of saturated maps. They have been extensively used to generate linkage maps in a number of plants such as soybean [7], peanut [8], *Catharanthus* [9], chickpea [10], pearl millet [11] and switchgrass [12]. In lentil, very few (about 200) genomic SSR markers have been developed and used for map construction [4, 13, 14] which are not enough for applications in lentil genomics. More recently a set of 122 new genomic SSR markers were reported by Verma et al. [15]. Their utilization in map construction would help to construct a more dense linkage map of lentil.

To date, no comprehensive SSR based intraspecific linkage map of lentil has been reported. However, with the advancement in sequencing and genotyping technologies SNPs have also been identified in lentil [16, 17]. In modern genetic analysis both the marker systems i.e. SSRs and SNPs have been found to be valuable for linkage mapping and QTL identification. Even though SNPs provide a number of advantages, SSRs are found to be more polymorphic and are considered as the best marker system for construction of framework linkage map [18]. Therefore it became imperative to isolate microsatellites from lentil and utilize them to construct a framework linkage map to identify QTLs for important agronomic traits.

Identification of QTLs for important agronomic traits has been made possible in a number of plant species with the availability of polymorphic markers and linkage maps. Seed traits, like seed size and seed weight are economically important quantitative traits, which are believed to be controlled by multiple genes. Seed size is a morphological trait and one of the important component of seed yield and the major target for breeding. A number of studies have been conducted to identify and map QTLs for seed weight/size in soybean [19], mungbean [20], *Jatropha* [21], *Brassica* [22] etc. Moreover, QTLs for seed traits, such as seed size and shape, have been identified in lentil also [23, 24]. Nevertheless, lack of dense linkage maps has limited their use for selecting stable QTLs for fine mapping.

Therefore, the present study was undertaken to construct microsatellite enriched genomic libraries of lentil for the generation of SSR makers to enrich the repertoire of polymorphic microsatellite markers. Next, these markers along with the other published SSR markers were used for construction of the linkage map of lentil using an intraspecific RIL mapping population (*L*. *culinaris* cv. Precoz x *L*. *culinaris* cv. L830). Further, phenotyping data for seed weight and seed size traits was utilized in order to identify QTLs for the same. The identification of QTLs controlling the agronomically important traits would enable to analyze association between the mapped loci and traits.

## Materials and Methods

### Ethics statement

The field site used for the study (CSK H.P. Agricultural University, Palampur and National Institute of Plant Genome Research, New Delhi, India) is neither privately owned nor protected and is meant for research purpose only for which no specific permission was required. The study did not involve any endangered or protected species.

### Plant material and DNA isolation

The intraspecific mapping population (RIL) of lentil was generated at the Department of Agricultural Biotechnology, CSK H.P. Agricultural University, Palampur. Briefly, for the generation of intraspecific recombinant inbred lines (RIL) two *Lens* genotypes contrasting for seed traits [*L*. *culinaris* cv. Precoz (macrosperma type, large seeds) and *L*. *culinaris* cv. L830 (microsperma type, small seeds)] were crossed and the resulting F_1_ plant was self-pollinated to obtain the F_2_ offspring that were further self-pollinated and advanced to F_8_ generation using single seed descent method to obtain RILs. An intraspecific mapping population comprising 126 RILs was used for the construction of linkage map and further for QTL analysis. All the plants were grown in natural conditions at the NIPGR field site during the growing season (Oct-April 2012–13).

Genomic DNA from fresh leaf tissue of all the 126 RILs of intraspecific mapping population along with the parental lines was isolated using a modified CTAB method as described previously [15]. The quality and quantity of all DNA samples was analyzed on agarose gels by comparison with known conc. of uncut λ DNA (25ng/μl).

### Phenotypic data evaluation

A total of 126 RILs derived from the cross between two lentil varieties *L*. *culinaris* cv. Precoz and *L*. *culinaris* cv. L830 were used for the construction of linkage map and QTL identification. The RILs were grown and phenotyped in the fields of CSK HP Agricultural University, Palampur during the growing seasons (Oct-April) of 2010–11, 2011–12 and 2012–13. The geographic coordinates for Palampur was 32.1167°N, 76.5333°E. The plants were grown in 2 meter long rows having row to row distance of 30cm and with a plant to plant distance of 5cm in a randomized block design (RBD) in three replications. The recommended agronomic practices were followed during the cropping season. The phenotypic data for seed weight and seed size traits for the RIL population was collected for 3 consecutive years. Data for each trait in the mapping population was measured using three replications. For average seed weight (g), a random sample of 100 seeds was taken for each individual plant. Seed size (mm) was determined by measuring size of seed (10 seeds in a row, covering the diameter of each seed) obtained from each plant sample and the average value was used for the analysis. Statistical analysis of the data, such as frequency distribution, variation coefficient and correlation coefficient analysis, was done using XLSTAT tool (http://www.xlstat.com/en/). The phenotypic correlation between these two traits was obtained using the Pearson’s correlations coefficient. These correlations were tested assuming global significance level of 0.05.

### Construction of a microsatellite enriched library and primer design

Two microsatellite enriched libraries of *L*. *culinaris* cv. Precoz was constructed using a biotin–streptavidin capture method as described earlier [15] for the identification of (GA)n and (GAA)n repeat motifs. The amplified fragments were ligated with the pGEMT vector and transformation was carried out using competent cells (NEB). After blue-white selection on IXA [isopropyl-b-D-thiogalactopyranoside (IPTG), X-gal and ampicillin] plates, the white recombinant clones were screened via colony PCR using M13 Forward and Reverse primers. The amplified products were resolved on 1.2% agarose gels to identify recombinants with inserts ≥250bp in size.

Plasmid DNA from the size selected clones was isolated using HiYield^**TM**^ Plasmid minikit [Real Biotech Corporation (RBC), Taiwan] and sequenced on the ABI 3700 Prism automated DNA sequencer using the Big Dye Terminator reaction kit (Applied Biosystems, USA). The sequences were subjected to Vecscreen followed by CAP3 program (http://pbil.univ-lyon1.fr/cap3.php). The resulting sequences were analyzed for the presence of microsatellites using the Gramene SSRtool http://www.gramene.org/db/markers/ssrtool. The flanking regions of the microsatellite containing sequences were used to design primers by the online software Primer 3 (http://frodo.wi.mit.edu/primer3/) using the criteria mentioned in Shrivastava et al. [25]. Primers were synthesized by Bioneer (Korea). The accession numbers were assigned to the microsatellite-containing sequences after submitting them to the NCBI database.

### Validation of microsatellite loci and electrophoresis

The markers generated in this study were validated by PCR amplification of Precoz genomic DNA in the Eppendorf (Germany) thermal cycler. Each PCR reaction was performed in 20 μl reaction mix containing 25 ng of DNA, 1X PCR buffer (50 mM KCl, 20 mM Tris–Cl pH 8.4), 1.5 mM MgCl_2_, 0.125 mM of each dNTPs, 0.5 μM of each primer, and 0.5U of *Taq* DNA polymerase (Life Technologies). The amplification was carried out using a touchdown amplification profile as described previously [26] and the amplified products were resolved on 8% polyacrylamide gels stained with ethidium bromide.

### Parental polymorphism and genotyping

For the identification of polymorphic markers, all the 501 markers generated in this study and 146 reported earlier were screened for polymorphism between the parents of the intra-specific mapping population (*L*. *culinaris* cv. Precoz and *L*. *culinaris* cv. L830). The 146 markers included 10 SSR series markers reported by Hamwieh et al. [13], 14 GLLC series markers reported by Saha et al. [27] and 122 LcSSR series markers reported by Verma et al. [15].

The polymorphic markers were further used for genotyping in the RILs by PCR amplification of genomic DNA as described above. The amplified products were resolved on 8% PAGE stained with EtBr and analyzed on Typhoon 9210 imager (Amersham Biosciences). The scoring was done based on the banding pattern observed on gel as ‘A’ representing *L*. *culinaris* cv. Precoz and ‘B’ representing *L*. *culinaris* cv. L830 and ‘H’ representing the heterozygote.

### Linkage analysis and map construction

A genotyping data matrix was generated based on the scoring pattern observed in the RILs with all the polymorphic genomic SSR markers. The matrix generated was integrated with the markers and used as an input file in Joinmap ver. 4.0 [28] for the construction of a framework linkage map. The locus genotyping frequencies of Joinmap was used to identify markers with aberrant segregation by performing Chi square test (*p <0*.*05*). Linkage groups were identified by grouping of markers at a minimum LOD threshold of 3 to a maximum of 8 with a step of 0.5. The groups were converted to maps at LOD 4 using the regression algorithm with recombination frequency smaller than 0.49, LOD >0.01 and performing a ripple after adding 2 loci. Kosambi’s mapping function was used to calculate map distance in centiMorgan (cM). Based on the positions and groupings of the SSR markers in the previously published maps of lentil [13, 27], the LGs were numbered accordingly.

### Statistical analysis and QTL mapping

Quantitative trait loci analysis for seed weight (SW) and seed size (SS) was carried out using the phenotypic data of 126 RILs using WinQTL Cartographer v2.5 [29]. In order to estimate the association of each marker to a trait, Single Marker Analysis (SMA) was performed followed by composite interval mapping (CIM) [30, 31] using the Zmapqtl standard model 6 with a window size of 10 cM and a 2 cM walk speed. In order to estimate a genome-wide LOD threshold score for QTL (P = 0.05), a 1000-permutation test was performed by shuffling the phenotypes means with the genotypes [32]. A LOD threshold score of >2.8 at 1000 permutations was considered significant to identify and map the QTLs on the lentil LGs. The 95% confidence intervals of the QTL locations were determined by one-LOD intervals surrounding the QTL peak [33]. The additive effect and the percentage of phenotypic variation explained by each putative QTL were estimated by the CIM method.

## Results

### Characterization of microsatellites and development of SSR markers

For the identification of microsatellites present in the lentil genome, two genomic libraries enriched for GA/CT (lib1) and GAA/CTT (lib2) were constructed using the nuclear DNA of *L*. *culinaris* cv. Precoz which yielded 2435 [1521 (lib1) + 914 (lib2)] recombinant clones. Screening through colony PCR resulted in the identification of 1616 [1014 (lib1) + 602 (lib2)] clones with insert size ≥250bp. All these clones were sequenced and 1103 [742 (lib1) + 361 (lib2)] clones with SSR motifs ≥3 were identified and were assembled into 758 [615 (lib1) + 143 (lib2)] consensus sequences using CAP3 and were used for primer design. However, primers could not be designed in 256 [205 (lib1) (33.3%)] and 51 [(lib2) (35.7%)] sequences due to lack of sufficient length of microsatellite flanking region. Eventually a total of 523 primers were designed from both the libraries [421 from (GA/CT) library + 102 from (GAA/CTT) library] and submitted to NCBI (accession numbers: JF768166-JF768701 and KJ470796-KJ470882). Of the total SSRs obtained, the targeted dinucleotides were the most abundant in the (GA/CT) library which constituted about 82.6% while in the (GAA/CTT) enriched library, the targeted motifs constituted 87.4%. Apart from the targeted ones, a number of other motifs were also obtained and used for primer designing. Perfect microsatellite motifs were identified in 65% (lib1) and 69.7% (lib2) sequences while the rest of the sequences consisted of interrupted [16.2% (lib1); 12.2% (lib2)] and compound repeats [18.8% (lib1); 18.1% (lib2)]. The range of the perfect GA/CT motif varied from 4 to 30 (LcSSR31 and LcSSR376) whereas for the perfect trinucleotide GAA/CTT, the motif number varied from 4 to 12 (LcSSR607). All the 523 primers generated in this study were validated in *L*. *culinaris* cv. Precoz. Five hundred and one primer pairs amplified products of the expected sizes and were designated as LcSSR markers (S1 Table). The remaining primers either didn’t amplify or produced anomalous fragments.

### Screening for parental polymorphism and genotyping of polymorphic markers in the mapping population

In the present study, a total of 647 lentil genomic SSR markers including 501 LcSSR series markers developed in this study, 122 LcSSR series markers reported by Verma et al. [15] and 24 genomic SSRs from previous studies in lentil (10 SSR series markers [13] + 14 GLLC series markers [27]) were used to identify polymorphic markers between the parental lines of the intra-specific mapping population contrasting for seed traits. Of the 647 genomic SSR markers 219 (33.9%) primer pairs [201 (32.3%) LcSSR series, 11 (78.6%) GLLC series, 7 (70%) SSR series] produced clear and consistent polymorphic bands between the parental lines and were used for genotyping in the 126 individuals. The genotyping data thus obtained was further used for the construction of linkage map. The total number of markers analyzed and the number of polymorphic markers has been listed in Table 1.

**Table 1:** Markers utilized for construction of the intra-specific linkage map of lentil (*L*. *culinaris* cv. Precoz x *L*. *culinaris* cv. L830).

Details of markers used in this study	Markers analyzed	Markers (% polymorphic)	Markers mapped
LcSSR (501 from this study + 122 from Verma et al. 2014)	623	201 (32.3%)	201
Hamwieh et al. 2005	10	7 (70%)	7
Saha et al. 2010	14	11 (78.6%)	11
**Total**	647	219 (33.8%)	216

### Construction of linkage map

**Fig 1:**
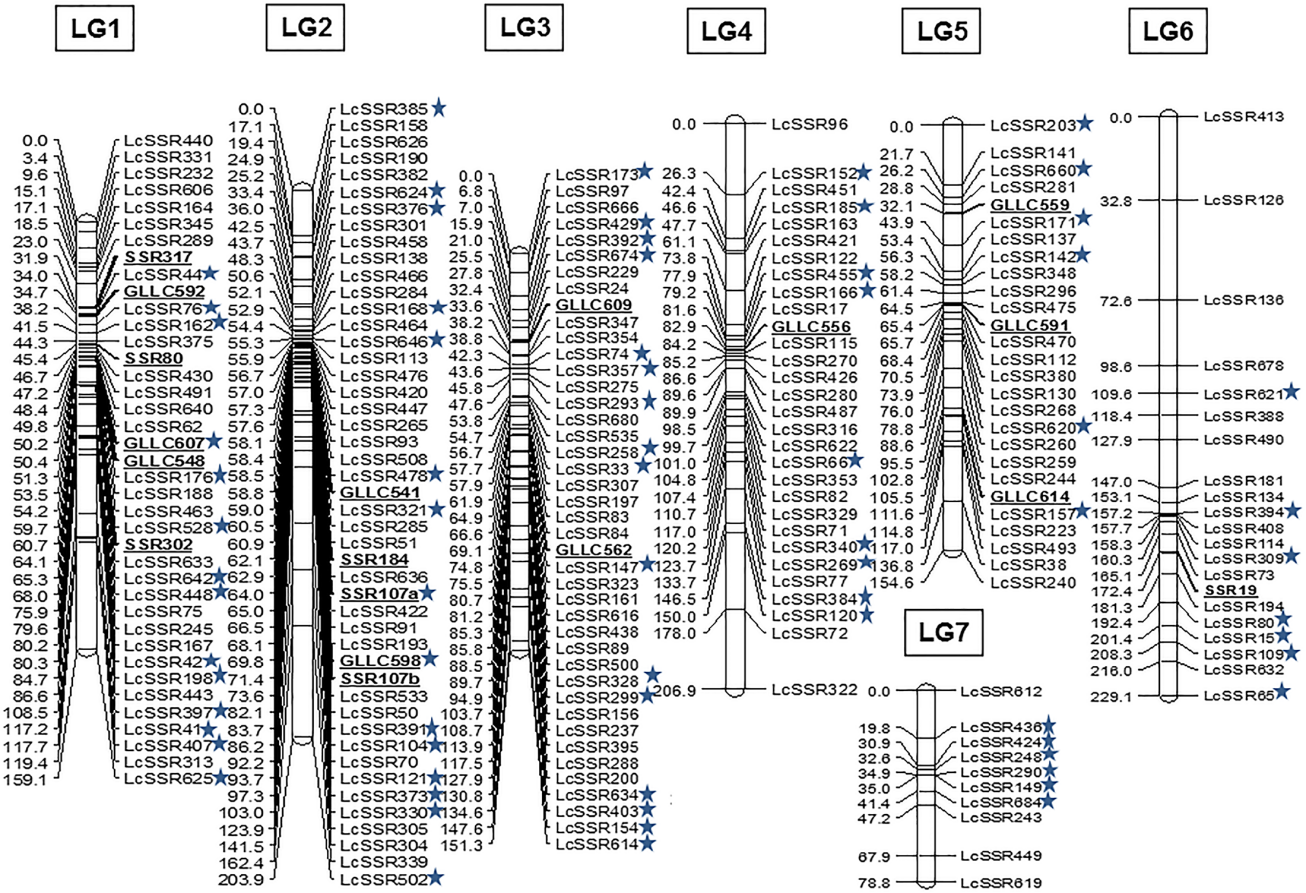
An intraspecific linkage map of *L*. *culinaris* based on RIL mapping population generated by crossing *L*. *culinaris* cv. Precoz× *L*. *culinaris* cv. L830. The map was generated with 219 polymorphic genomic SSR markers using JoinMap version 4.0 at a LOD value of 4.0, with Kosambi mapping function. A total of 216 markers were mapped on seven linkage groups (LGs) (LG1 to LG7). The previously mapped markers used in this study are underlined. The distorted markers have been marked with “*” on the right side of the marker name.

An intraspecific genetic linkage map of lentil was constructed using the genotyping data (S2 Table) of 219 polymorphic markers using the JoinMap software. At LOD 4.0, 216 markers among 219 polymorphic markers were assigned positions on seven linkage groups (LGs). The intra-specific map generated here spanned 1183.7 cM distance of the lentil genome with an average marker density of 5.48 cM (Fig 1). The 216 mapped markers included 174 LcSSR series (generated in this study and published earlier), 7 markers of SSR series and 11 markers of GLLC series. Each linkage group differed from each other with respect to total number of markers mapped, total cM distance and marker density (Table 2). A large variation in length was exhibited by these seven linkage groups which varied from a minimum of 78.8 cM (LG7) to a maximum length of 229.1 cM (LG6). The average marker density on each linkage group revealed that the markers were distributed randomly and unevenly (Fig 1). The marker density was highest in LG3 (3.6cM) which harbored 47 markers, and lowest in LG6 (10.91cM) with 21 markers.

**Table 2:** Distribution of the 216 genomic SSR markers on the seven linkage groups of an intra-specific linkage map of lentil (*L*. *culinaris* Precoz x *L*.*culinaris* L830).

LGs	Markers mapped	Map length (cM)	Average marker density (cM)	Skewed markers (%)
**LG1**	39	159.1	4.1	14(35.9)
**LG2**	47	203.9	4.3	15(31.9)
**LG3**	42	151.3	3.6	16(38.1)
**LG4**	30	206.9	6.9	9(30)
**LG5**	27	154.6	5.8	6(22.2)
**LG6**	21	229.1	10.9	7(33.3)
**LG7**	10	78.8	7.9	6(60)
**Total**	**216**	**1183.7**	**5.5**	**73(33.8)**

About 18 markers, reported by Hamwieh et al. [13] and Saha et al. [27] were located on the map generated in this study. Based on the positions of these common markers, the map was compared to the previously published maps. LG1 spanned 159.1 cM, which harbored 33 LcSSR markers and shared 3 common markers (SSR317, SSR80 and SSR302) with LG1 of Hamwieh et al. [13] and 3 common markers (GLLC592, GLLC607 and GLLC548) with LG1 of Saha et al. [27]. LG2 harbored 47 loci including 3 common loci (SSR184, SSR107a and SSR107b) with LG2 of Hamwieh et al. [13] and 2 common loci of GLLC series (GLLC541 and GLLC598) with LG11 of Saha et al. [27]. LG3 defined positions of 40 loci of LcSSR series and 2 common markers (GLLC562 and GLLC609) with LG2 of Saha et al. [27] and was the most densely populated group with average marker density of 3.60 cM. LG4, which comprised of 30 markers spanning 206.9 cM, shared only 1 common marker (GLLC556) with LG4 of Saha et al. [27] whereas LG5 containing 27 markers shared 2 common markers (GLLC559 and GLLC591) with LG12 of the same map. LG6 shared only one common marker (SSR19) with the map of Hamwieh et al. [13].

Segregation distortion for all the 219 polymorphic markers was calculated among which 146 (66.2%) followed the expected segregation ratio, whereas 73 loci (33.8%) were found to show deviation. Thirty eight markers (52.1%) among 73 loci showed slight deviation and were skewed towards the parent *L*. *culinaris* cv. Precoz while 35 loci (47.9%) exhibited significantly high segregation distortion from the expected Mendelian ratio (1:1) and were skewed towards *L*. *culinaris* cv. L830. The degree of skewness in these loci varied significantly. Further, all of the distorted loci, were mapped and mostly resided on LGs 3, 2 and 1 (Fig 1).

### Phenotypic variation in parents and mapping populations

**Fig 2:**
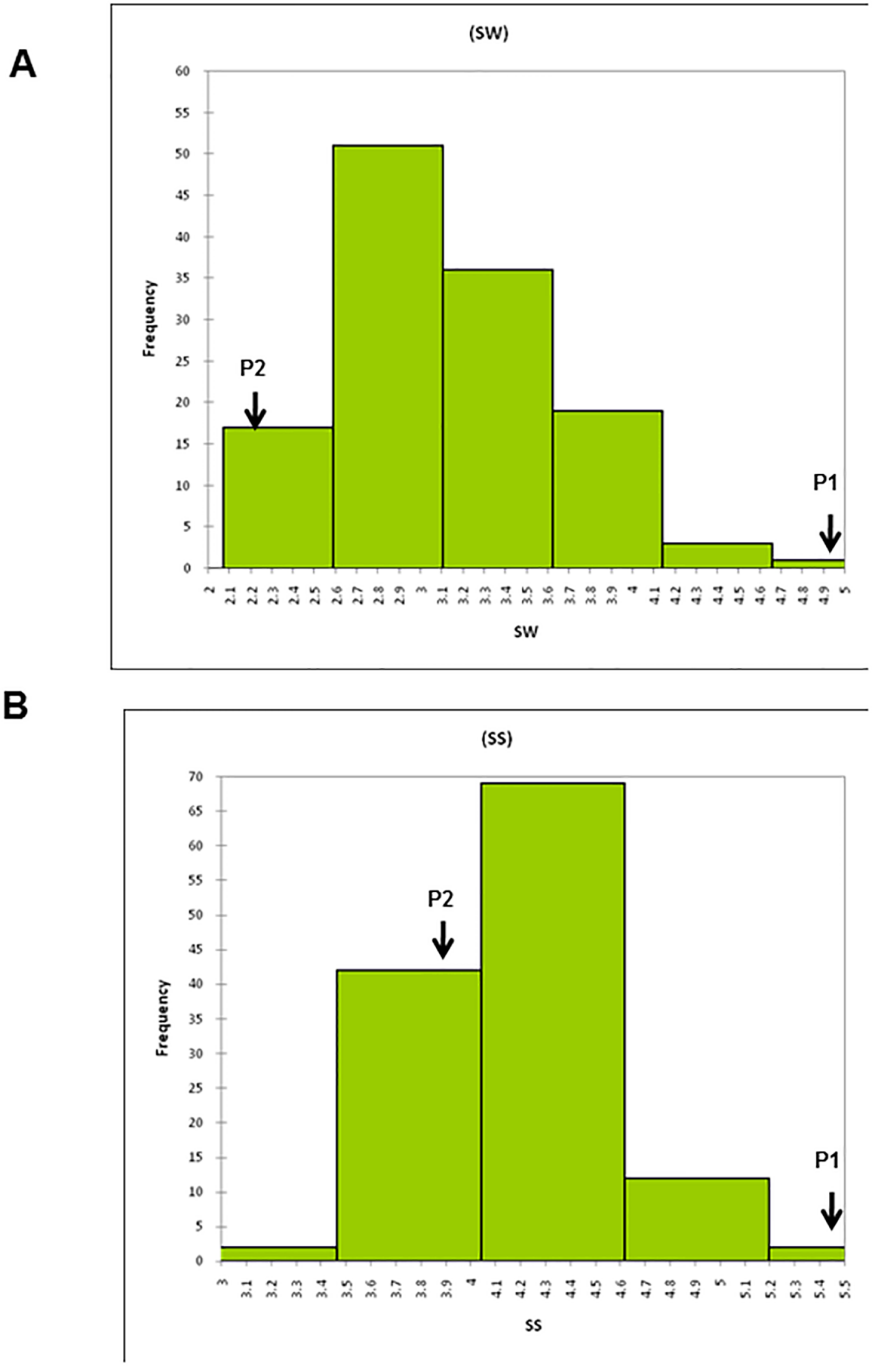
Frequency distribution of A. SW (seed weight in g) and B. SS (seed size in mm) phenotypic traits for the 126 RILs as depicted by the histogram. The phenotype of both the parents P1 (*L*. *culinaris* cv. Precoz) and P2 (*L*. *culinaris* cv. L830) are indicated by arrows.

Agronomic data was collected for the two seed traits in the segregating RILs including the parents of the mapping population (Precoz and L830) which revealed that they are highly contrasting for 2 morphological seed related traits which ranged from 2.1–4.3g for seed weight and from 3.2–5.2mm for seed size (Table 3). The individuals also exhibited a variation for these traits as revealed by their mean values. The phenotypic variations among the 126 RIL lines were obvious in all the three years and the frequency distributions of all traits in the progeny showed a continuous distribution (S3 Table). The means of the parental lines and the frequency distribution of the RILs for each trait are shown in Fig 2. The two traits showed a positive Pearson’s correlation with a P-value of 0.76.

**Table 3:** Descriptive statistics of phenotype data of mapping population and parents (*L*. *culinaris* cv. Precoz and *L*. *culinaris* cv. L830).

Trait	Precoz	L830	Min.	Max.	Mean	Variation coefficient	Std. deviation
SW (g)	5.1	2.2	2.1	4.3	3.1	0.2	0.5
SS (mm)	5.7	3.9	3.2	5.2	4.2	0.1	0.4

‘Min’ and ‘Max’ indicate the values of the RILs with the lowest and the highest value respectively. ‘Mean’ is the average value for all RIL lines. SW (g) = 100-seed weight and SS (mm) = Seed size (10 seeds).

### Mapping of seed related traits

**Fig 3:**
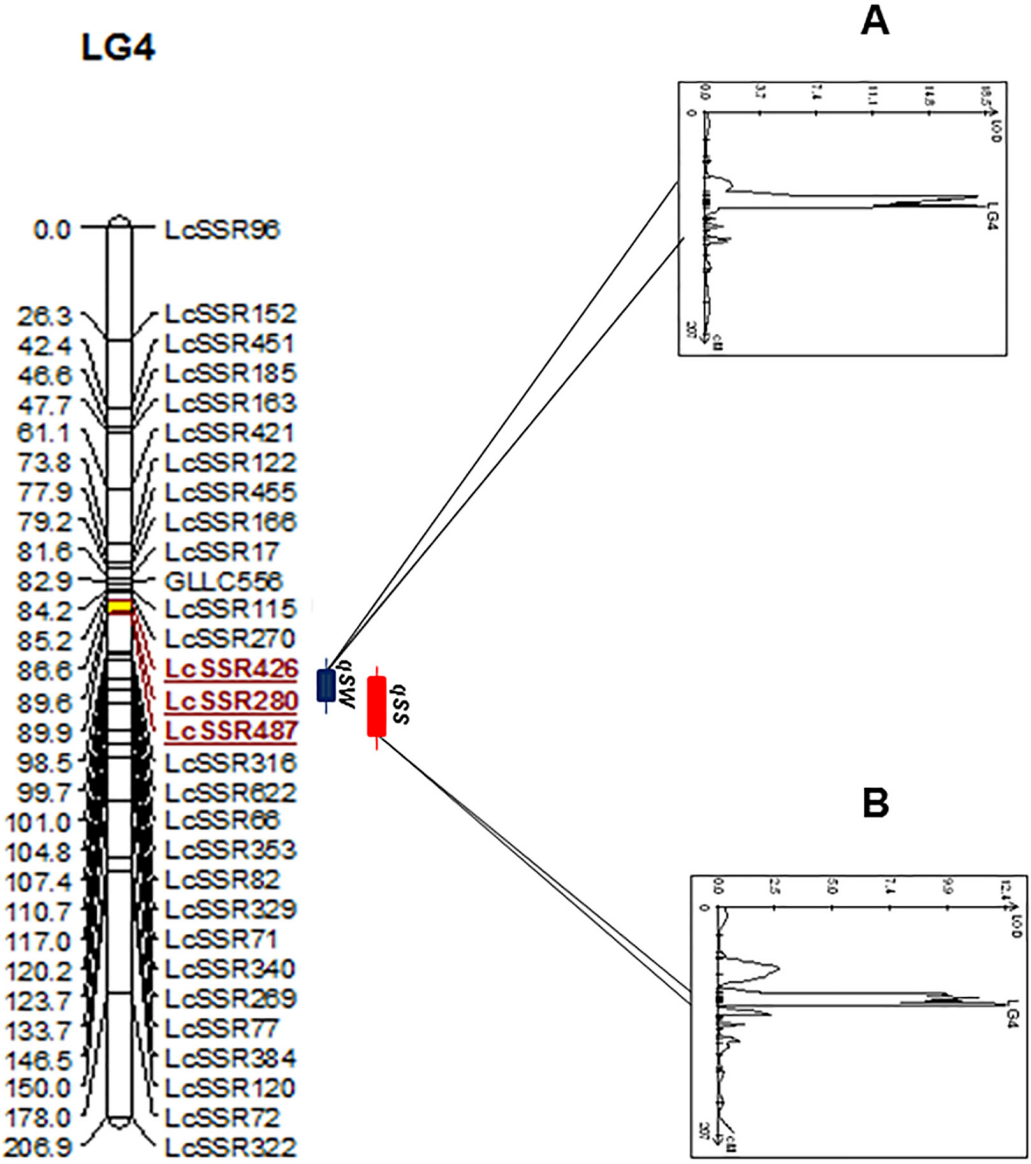
Chromosomal locations of detected QTL. LOD peak value of ≥2.8 was considered to indicate a significant QTL interval. The QTLs are indicated by rectangular bars of different shades in the right side of the linkage groups followed by the name of the QTL. The LOD score plot of individual trait [(A) seed weight and (B) seed size] is displayed on the right side of the bars locating the marker positions. The region of identified QTLs on the linkage group is highlighted by yellow color.

QTLs for the two seed traits were identified by WinQTL Cartographer v2.5 using the phenotypic data of the RILs collected for three consecutive years. Three major QTLs (*qSW1*, *qSW2*, *qSS1)* were detected by SMA for both the traits on LG4. The QTLs *qSW1* and *qSW2* were identified at LOD score of 13.3 and 14.3 respectively with positive additive effects. Similarly the QTL *qSS1* was identified at LOD score of 15.2 with positive additive effect. Further, using the CIM method two major QTLs, one for each trait were detected and were co-localized on the same linkage group i.e. LG4 with LOD score greater than their corresponding permutation threshold (LOD ≥ 2.8) (Table 4). The QTL LOD plot of the individual traits has been depicted in S1 Fig with their highest peak values and threshold values. These QTLs explained 27.5% and 48.4% of the phenotypic variance for seed size and seed weight respectively with positive additive effects indicating that the alleles from Precoz increased these trait values. Among the two QTLs, *qSW* contributed the most with LOD score of 18.5 and phenotypic variance of 48.4% (Table 4) and was flanked by LcSSR426 and LcSSR280 as the interval markers that were 3.0cM apart. The QTL *qSS* spanned an overlapping region of 3.3cM flanked by LcSSR426 and LcSSR487. In the region of markers associated with these traits on LG4, the LOD-score plots could not separate the two seed related QTLs indicating a tight association of these traits. (Fig 3).

**Table 4:** QTLs identified for two seed traits in Precoz x L830 mapping population.

Trait	QTL	LG	Marker Interval	CI(cM)	QTL Pos.	LOD	Additive effect^a^	Phenotypic variance (%)
Seed weight	*qSW*	4	LcSSR426-LcSSR280	86.6–89.6	88.6	18.5	0.3	48.4
Seed size	*qSS*	4	LcSSR426-LcSSR487	86.6–89.9	88.6	12.4	0.2	27.5

QTL: starting with “q,” followed by an abbreviation of the trait name.

^a^: Positive values indicated additive effects contributed by the alleles of one parent (Precoz)

## Discussion

Genetic linkage maps are important for several applications in genetics research and plant breeding such as mapping of desirable traits and QTL identification. The availability of large numbers of genetic markers is the first step towards the construction of linkage map and mapping of traits of interest. Microsatellite markers have proven to be highly efficient for the construction of linkage maps and molecular analysis in plants [34]. They have been considered as an important genomic resource for plant breeders and have been extensively used for a wide range of applications [35]. Though SSR markers are important molecular tools, their critical mass required for developing a dense genetic map, is still limited in lentil. Microsatellite markers have been developed and reported in lentil by Hamwieh et al. [13, 14], Verma et al. [15] and Závodná et al. [36], but very few of them have been used in mapping till date. Therefore, for the construction of a high density linkage map of lentil, more markers were required which necessitated this study.

A number of enrichment methods have been extensively used for the large scale isolation of microsatellites in plant genomes [37, 38] and each technique differs from the other in terms of cost, time and labor. In this study two genomic libraries enriched for (GA)n and (GAA)n repeats were constructed as GA/CT motifs are the second most abundant dinucleotides in plants after AT/TA repeats [39] and GAA/CTT repeat motifs are the most abundant among trinucleotides [40]. The enrichment efficiency was quite high [73.2% (lib1) and 60% (lib2)] and comparable to the previous reports of lentil where enrichment varied from 71.1% [14] to 73% [15].

The screening of library revealed that 73.2% (lib1) and 60% (lib2) of recombinant clones from both the libraries contained microsatellite motifs and about 66.2% of them were ultimately found suitable for primer designing. As a result 523 SSR markers were designed from both the libraries. The validity of the primers designed was evaluated by their efficiency to amplify the target sequence. All the primers designed were validated in the parent DNA (*L*. *culinaris* cv. Precoz) and among them, 95.8% amplified products of expected sizes which was significantly higher than the amplification efficiency reported earlier in lentil (67.1%; [14], 80.8%; [15]) and other plants like eggplant (75.3%; [41], switchgrass (73.7%; [42]) and *Catharanthus* (91.8%; [9]).

In the present study, 647 genomic SSR markers (501 markers developed in this study from both the libraries + 122 LcSSR series; [15] + 14 GLLC series; [27] + 10 SSR series; [13]) were screened for polymorphism between the parents of the mapping population used in this study. Of these, 219 (33.9%) were found to be polymorphic which was higher than the genomic SSRs previously reported in lentil (24.2%, [13]; 4.4%, [43]) and other legumes such as pea (15.8%; [44]), chickpea (22.1%; [45]), peanut (23.6%; [8]), azuki bean (26.8%; [46]), soybean (27%; [7]) and lotus (37%; [47]). It has been documented that different molecular tools for genomic analysis and improvement could not be extended in legumes beyond a certain level due to their narrow genetic base [43]. A number of factors affect the level of polymorphism exhibited by a mapping population such as type of marker, type of cross (self- or cross-pollinated, inter- or intraspecific cross), type of population (F_2_/BC/RIL) etc. Crossing within the cultivated species are preferred for breeding as they identify polymorphic markers within the cultivated gene pool [48] and may also minimize the problem of linkage drag which is often observed in the crosses involving wild species [49].

The present linkage map defined positions of 216 polymorphic genomic SSR markers which were distributed on 7 linkage groups spanning 1183.7 cM distance with an average marker density of 5.48 cM. The map length was comparable to other intraspecific linkage maps of lentil (784.1 cM; [50], 1192 cM; [51], 1868 cM; [52], 1565.2 cM; [27]) with the added advantage of being denser than these maps. Among the 7 linkage groups, each group differed from the other with respect to length and marker distribution as a result of which some groups were densely populated (LG1 and LG3), while some exhibited few markers (LG7 and LG6) which could be explained by the fact that SSRs are ubiquitously and randomly distributed in plant genomes. Most of the markers were located on the centromeric region, may be due to the lower recombination frequency in these regions [53, 54]. The genomic origin of DNA sequences used for the SSR identification was also responsible for their unequal distribution on the groups and thus lead to less genome coverage [55].

Segregation distortion is a common feature which is observed in most of the linkage maps in plants and has been attributed to difference in DNA content, structural rearrangement, self-incompatibility and deleterious recessive alleles [56–59]. In the present study, significantly high distortion was exhibited by 33.3% of the markers. Different levels of segregation distortion in lentil mapping populations have been observed (9.5% & 17.8%, [13]; 14.4%, [50]; 12%, [60]; 20%, [52]; 48%, [61]; 22%, [27]; 23.8%, [62]). Of all the types, highest distortion is exhibited by the RILs [63]. However, this is not always true as about 83.3% of segregation distortion has been observed in previous lentil map using F_2_ population Eujayl et al. [64]. Thus, it may be inferred that population type is not the only factor which is responsible for distortion but other factors such as chromosome loss during the process of crossing over [65], isolation mechanisms [66] or the presence of other alien or viability genes [67, 68].

The availability of genetic maps allows the localization and mapping of different agronomically important traits with the help of phenotypic data of segregating populations. Seeds are an important component of grain legumes. Mapping of QTLs related to seed traits can enable dissection of their genetic control and molecular mechanism which can open the possibility to develop varieties with improved seed quality and enhanced seed yield. In the present study, the RIL population of an intraspecific mapping population (Precoz x L830) was evaluated for 2 seed traits (seed weight and seed size). Using SMA, a total of three QTLs were detected for both the traits. But using CIM, two major QTLs for both the traits were detected. The two major QTLs for both seed weight and seed size, detected on LG 4 explained 48.4% and 27.5% of phenotypic variation respectively. The QTL region for both the traits was small (3.3cM) leading to fairly good resolution of all the identified QTLs. Further fine mapping may help to narrow down the QTL region to a smaller region. QTLs for seed weight and seed size in lentil have been previously mapped by Fratini et al. [24]. The phenotypic variance observed for seed weight in our study was higher (48.4%) than the QTLs identified by Fratini et al. [24] on LGI, LGIII and LGVI (total phenotypic variance: 18.2%). However the phenotypic variance of 37% and 60% was reported by Fratini et al. [24] and Fedurok et al. [69] for seed size QTLs which was higher than that reported in our study (27.5%). Tahir and Muehlbauer [70] also located three QTLs for seed weight on linkage groups 1, 4 and 5, respectively. Since no consensus linkage map of lentil is available, the comparison of QTLs identified from the previous works is difficult with respect to their map position.

The QTLs detected for the two traits were co-localized on LG4 and spanned 11.7cM region. QTL clusters having more than one trait is a common occurrence and the related traits may have multiple effects on each other as they belong to the same genomic region. The clustering of QTLs can arise due to pleiotropic effect of a single regulatory gene [71]. The occurrence of pleiotropy could be explained in a way that certain traits are phenotypically correlated with each other due to the presence of certain genes coexisting in these QTLs. Fine mapping of these identified QTLs would provide a better picture to understand whether linkage or pleiotropic effects are responsible for their clustering.

## Conclusions

Overall, SSR markers and in particular the linkage maps are extremely useful for plant breeding and crop improvement programs, wherein they have profound applications. Evidently, the map generated in the present study although covered a significant portion of the genome, a further saturation of this map with additional markers (SSRs or SNPs) is imperative for its efficient utilization. With the long-term goal of understanding the genetic basis of seed weight and seed size, the present study was focused on identification of major QTLs for both the traits in lentil. A SSR-based linkage map was constructed to facilitate this type of study. In conclusion, we envisage that the present linkage map, fortified with 216 SSR markers, and QTLs for seed size and seed weight would provide a means to breeders for further enhancement of the crop. The knowledge of marker-trait association may lead to the identification of genes influencing agronomic traits. Therefore, the SSR based linkage map and QTL mapping is an effort to provide a basis for MAS in future lentil breeding programs.

## Supporting Information

S1 Fig Quantitative trait locus (QTL) likelihood plot associated with (A) seed weight and (B) seed size identified near the position of marker LcSSR426-LcSSR487 on LG4 (C) The cluster of both the traits is identified on the same plot. The vertical axis indicates the LOD score, and the horizontal axis indicates distances in cM based on composite interval mapping. The bar above *x axis* indicates the peak QTL region. The horizontal line indicates the LOD threshold (≥2.8) for both the traits which was empirically determined by performing 1000 permutations of the data.
(PPT)

S1 Table **A**. List of *L*. *culinaris* SSR primer pairs developed from the (GA)_20_ enriched microsatellite library. The primer sequences (F/R), microsatellite repeat motif structure, annealing temperature (Tm), expected size of the amplification product (bp), along with the GenBank accession numbers are mentioned. **B**. List of *L*. *culinaris* SSR primer pairs developed from the (GAA)_14_ enriched microsatellite library. The primer sequences (F/R), microsatellite repeat motif structure, annealing temperature (Tm), expected size of the amplification product (bp), along with the GenBank accession numbers are mentioned.
(DOC)

S2 Table Genotyping profile of lentil RIL mapping population used to generate linkage map. (XLS)

S3 Table Average values of the three years phenotyping data for the two seed traits (SW: seed weight and SS: seed size). (XLS)
