# Supplementary figures and images for: Construction of a Genetic Linkage Map and Identification of QTLs for Seed Weight and Seed Size Traits in Lentil (Lens culinaris Medik.)
Source: PLoS One. 2015 Oct 5;10(10):e0139666. doi: 10.1371/journal.pone.0139666 (PMC4593543; doi:10.1371/journal.pone.0139666)

## Slide 1
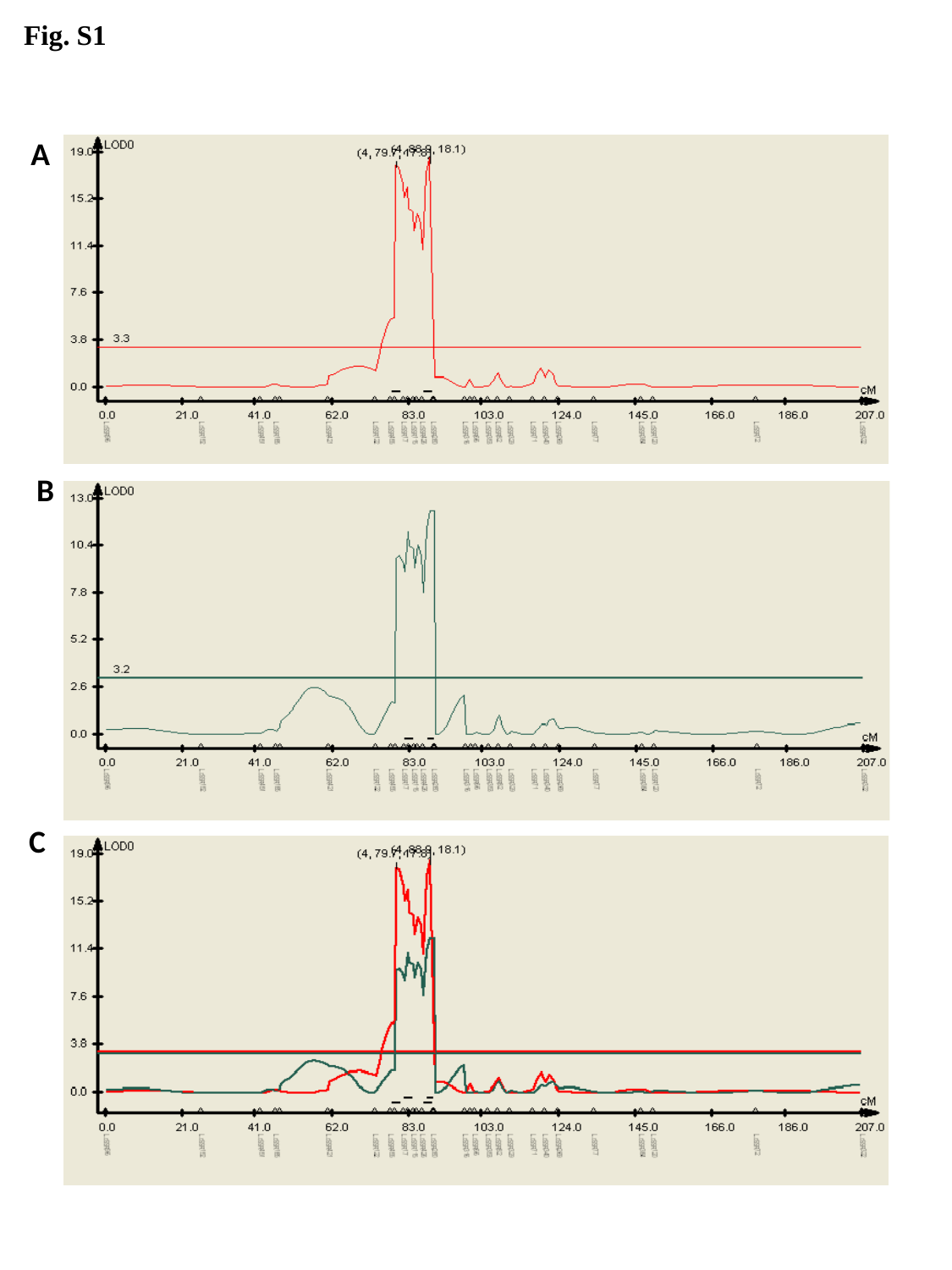

Fig. S1
A
B
C

Supplement: S1 Fig — The vertical axis indicates the LOD score, and the horizontal axis indicates distances in cM based on composite interval mapping. The bar above x axis indicates the peak QTL region. The horizontal line indicates the LOD threshold (≥2.8) for both the traits which was empirically determined by performing 1000 permutations of the data. (PPT) [file pone.0139666.s001.ppt]
