# Supplementary material for: Construction of a Genetic Linkage Map and Identification of QTLs for Seed Weight and Seed Size Traits in Lentil (Lens culinaris Medik.)
Source: PLoS One. 2015 Oct 5;10(10):e0139666. doi: 10.1371/journal.pone.0139666 (PMC4593543; doi:10.1371/journal.pone.0139666)
Supplement: S1 Table — A. List of L. culinaris SSR primer pairs developed from the (GA)20 enriched microsatellite library. The primer sequences (F/R), microsatellite repeat motif structure, annealing temperature (Tm), expected size of the amplification product (bp), along with the GenBank accession numbers are mentioned. B. List of L. culinaris SSR primer pairs developed from the (GAA)14 enriched microsatellite library. The primer sequences (F/R), microsatellite repeat motif structure, annealing temperature (Tm), expected size of the amplification product (bp), along with the GenBank accession numbers are mentioned. (DOC) [file pone.0139666.s002.doc]

**Construction of a genetic linkage map and identification of QTLs for seed weight and seed size traits in lentil (*Lens culinaris* Medik.)**

**Priyanka Verma^1,3^, Richa Goyal^1^, RK Chahota^2^, Tilak R Sharma^2^ , M Z Abdin ^3^ and Sabhyata Bhatia^1^***

^1^National Institute of Plant Genome Research, Post Box No. 10531, Aruna Asaf Ali Marg, New Delhi-110067, India

^2^Department of Agricultural Biotechnology, Chaudhary Sarwan Kumar Himachal Pradesh Agricultural University, Palampur 176 062, India

^3^Department of Biotechnology, Faculty of Science, Jamia Hamdard, New Delhi-110062, India

***For Correspondence: E-mail:** [sabhyatabhatia@nipgr.ac.in](mailto:sabhyatabhatia@nipgr.ac.in)

**Phone:** +91 011 26735159

**Fax:** +91 011 26741658

**S1A Table. List of *L. culinaris* SSR primer pairs developed from the (GA)_20_ enriched microsatellite library. The primer sequences (F/R), microsatellite repeat motif structure, annealing temperature (Tm), expected size of the amplification product (bp), along with the GenBank accession numbers are mentioned.**

| **S.No.** | **Locus** | **Sequence**  **F: Forward (5’-3’)**  **R: Reverse (5’-3’)** | **Repeat motif** | **Tm**  **(ºC)** | **Product Size (bp)** | **Accession No.** |
| --- | --- | --- | --- | --- | --- | --- |
| 1 | LcSSR1 | F: GTTTGGTCCGGTTCACCTTA  R: TAACCATCGTCGGATTCTGG | (CT)_9_ | 60 | 284 | JF768166 |
| 2 | LcSSR2 | F: TAAAAGACGGTGGCGACTCT  R: ACGTCGATATCGGCTGAAAC | (CT)_18_…(AT)_5_ | 60 | 367 | JF768167 |
| 3 | LcSSR3 | F: TTTTGACAGCTCGGTTGTGT  R: TTTTCGTTAATCCGCAGAAA | (GA)_9_ | 59 | 377 | JF768168 |
| 4 | LcSSR4 | F: GGATGGAGTTTCAGCTTCCTT  R: GCTTTTCCAATCTCAGCTATGC | (GA)_7_ | 60 | 227 | JF768169 |
| 5 | LcSSR5 | F: AGGGTTTGACTCGCTGAAGA  R: TACCAATCCAAAGGCCATGA | (GA)_9_ | 61 | 284 | JF768170 |
| 6 | LcSSR6 | F: CAAAATCGTTCTCATAGGGAAAA  R: GGATTAGTAGTTATTGTTGGAGATACC | (CT)_19_ | 59 | 238 | JF768171 |
| 7 | LcSSR7 | F: TTTTCAACGGCTCCTTTGTT  R: TTGCTTCAAAAAGCTATATCACAGA | (CT)_11_ | 59 | 263 | JF768172 |
| 8 | LcSSR8 | F: CAAAGGCACAAACGACAAAA  R: CAAAGACTATCAGAAAGACGTTCAA | (GA)_8_ | 59 | 323 | JF768173 |
| 9 | LcSSR9 | F: CGACATGACGGTTCATCAAT  R: CAAACATTACAAAATAATGCAACAGC | (GA)_10_ | 60 | 298 | JF768174 |
| 10 | LcSSR10 | F: ACCTCATTTTTCCACGTTCG  R: TCTTTCCTTGCCCTAGTCCT | (GA)_4_…(GA)_9_ | 59 | 273 | JF768175 |
| 11 | LcSSR11 | F: CCGAACAAATTTTCCCCTTA  R: GCGTGGAAGTCGGTAAAAGA | (GA)_12_ | 60 | 260 | JF768176 |
| 12 | LcSSR12 | F: TGCCTATGTTGTTGTTGTGATG  R: GCCAAGTTATCGAGGCATCT | (GTT)_3_…(GTT)_4_ | 59 | 298 | JF768177 |
| 13 | LcSSR13 | F: TCCACCAATAGTGCACATGAA  R: AAGGGGTATGTCGGTCATTG | (CAA)_2_t(CAA)_3_…(CAA)_3_…(CAT)_3_..(CT)_18_ | 59 | 405 | JF768178 |
| 14 | LcSSR14 | F: TTGCAAGATGACGTTGCTGT  R: GCCACTACCAAACATGGAACA | (GA)_11_…(AT)_4_ | 60 | 287 | JF768179 |
| 15 | LcSSR15 | F: CAATGTTTGTGTATGAGATTGCTTC  R: AAATTCAAGAGAACAAAAACTACGC | (GAT)_3_…(CTT)_4_…(GA)_10_ | 59 | 282 | JF768180 |
| 16 | LcSSR16 | F: TCCAGAAAAAGGGCTACAGAA  R: CGCCTCTCTTTGATTTGCTA | (CT)_8_ | 58 | 230 | JF768181 |
| 17 | LcSSR17 | F: GGGTTCATAAATCCCCTTTCA  R: ATAAGTTTCGGGGTTTAGGG | (GA)_11_..(GT)_3_ | 60 | 219 | JF768182 |
| 18 | LcSSR18 | F: AAGTGTATTCTTGGCATTTGGAA  R: GCCACGACAACAGTGAGTTT | (AT)_3_…(AT)_3_…(CT)_7_ | 59 | 278 | JF768183 |
| 19 | LcSSR19 | F: GAAGCATGGTGTTGAAGCAA  R: TCCCAAAACATTTCGTCCTC | (GA)_14_ | 59 | 281 | JF768184 |
| 20 | LcSSR20 | F: TTAATGATGCGCAATGGCTA  R: AGAAGGATATTTCTCCCAAAAGA | (CT)_8_ | 60 | 264 | JF768185 |
| 21 | LcSSR21 | F: GCTTCAAAAAGCTTTATCACAACA  R: TGGGAGTTTTCCAAGACAGAA | (CTT)_4_…(GA)_13_ | 59 | 268 | JF768186 |
| 22 | LcSSR22 | F: CGACCTGGGATTCATTATCCT  R: TCTTCCAAATTTAAAGTAAAGCTTCC | (CA)_4_…(GA)_9_ | 60 | 250 | JF768187 |
| 23 | LcSSR23 | F: GCAAAAGTGGGGTGTAAAGG  R: GATGGATGCGTTGGGTAAAG | (CT)_3_cg(CT)_5_…(CT)_4_ | 60 | 383 | JF768188 |
| 24 | LcSSR24 | F: TCGACAGAGTCGAATACAACAT  R: TGTTGTGTTACAGTTTGCCATT | (CT)_17_…(CTT)_3_…(GA)_3_ | 58 | 317 | JF768189 |
| 25 | LcSSR25 | F: GGATCTGAGACTGAGACAAAACA  R: CTTTTTGGGCACAATTCCTC | (GA)_8_ | 59 | 292 | JF768190 |
| 26 | LcSSR26 | F: TTTGATGATGATCAGTTGTTCTTG  R: AGCAAACATTCTTTTGGCATC | (CTT)_4_…(GA)_12_ | 59 | 129 | JF768191 |
| 27 | LcSSR27 | F: TCACAAACTGTGATGTTTTACACAA  R: TGGGAGTTTTCCAAGACTGA | (CTT)_5_…(GA)_9_…(GTT)_3_…(GTT)_4_ | 59 | 258 | JF768192 |
| 28 | LcSSR28 | F: TTTCAATTTTGCTCTCTCTTTTG  R: TTTTGCATGTAACGTGATTGG | (CT)_9_ | 59 | 247 | JF768193 |
| 29 | LcSSR29 | F: TCTCGAATAAAGGAGATTTGGTG  R: TCTTTTACCTTTGGTGTCTTCG | (AT)_5_…(GA)_14_ | 59 | 416 | JF768194 |
| 30 | LcSSR30 | F: CGGATCCTTTGTAGAAGAAAGA  R: ACAACCGTGGTCACTCAAGA | (CTTT)_2_…(CT)_7_…(GAA)_4_ | 59 | 348 | JF768195 |
| 31 | LcSSR31 | F: CTTTCATAAACCGCCCTTGC  R: AAAGACGGTGGCGACTCTTA | (GA)_30_ | 61 | 329 | JF768196 |
| 32 | LcSSR32 | F: CAAAATCGTTCTCATAGGGAAAA  R: TTGATCCAACATCGAAGCAT | (CT)_28_ | 59 | 279 | JF768197 |
| 33 | LcSSR33 | F: TGCAATAAATCGCTACCAATAGAA  R: GAAATCATATTGGAAGACTAACGAG | (GT)_5_…(GA)_16_…(AT)_3_ | 60 | 253 | JF768198 |
| 34 | LcSSR34 | F: TGGGAGTTTTCCAAGACAGAA  R: TGGTTCGTTGACCAAACTGT | (CT)_9_…(ATT)_3_ | 59 | 271 | JF768199 |
| 35 | LcSSR35 | F: GATGAAGATCAGATTGTTCTTGTAAA  R: AAAGGTGTTTACCATGCTGCT | (CTT)_4_…(GA)_17_…(CTT)_4_ | 58 | 332 | JF768200 |
| 36 | LcSSR36 | F: GGAACAATGCAATAAACCTGCT  R: TGGATCAAGTGGTATATTTGGACA | (CTT)_4_…(GA)_10_…(GTT)_4_ | 60 | 339 | JF768201 |
| 37 | LcSSR37 | F: CTCAAGGAACAAACCGCAAT  R: TGGGAGATTTTCCAAGACACA | (CTT)_3_…(GA)_7_ | 60 | 376 | JF768202 |
| 38 | LcSSR38 | F: GGGTGAAAAGAAACTACGCTGA  R: AAAACAACAACACTGACGAATCA | (AAT)_3_…(GATA)_2_…(GA)_7_ | 60 | 266 | JF768203 |
| 39 | LcSSR39 | F: TTGATGAAGATCAGAGTGTTCTTGT  R: TTGAGGGAGATTTCCAAGAAAG | (CTT)_4_…(GA)_8_gg(GA)_4_ | 59 | 249 | JF768204 |
| 40 | LcSSR40 | F: GCTTCTAAAAAGCTTTATCACAAACTG  R: GCCTTTTCAACGGCTACTTT | (CTTT)_3_…(GA)_9_ | 60 | 301 | JF768205 |
| 41 | LcSSR41 | F: GCTTCAAAAAGCTTTATCACAAAAG  R: TGGGAGATTTCCAAGACTGG | (GA)_9_ | 60 | 267 | JF768206 |
| 42 | LcSSR42 | F: GCTTCTAAGAAAGCAGAATCACAA  R: TGGCATCAAGAATATTAACAACA | (GTT)_3_…(GA)_15_ | 59 | 209 | JF768207 |
| 43 | LcSSR43 | F: GCTTCAAAAAGCTATATCACAACA  R: GCCTTTTCAACGGCTATTTT | (CTT)_3_…(GA)_12_ | 58 | 312 | JF768208 |
| 44 | LcSSR44 | F: GCACACATCACAGCAAATCC  R: CCGATTCATATATTTTGCCTGA | (CTT)_4_…(CTT)_3_…(CTT)_3_…(CTT)_6_ | 60 | 305 | JF768209 |
| 45 | LcSSR45 | F: GCTGTATCACTAACCGTGATGTTTT  R: GTCAGCACACGATTGGACAT | (CTT)_3_…(GA)_7_…(CTT)_3_ | 60 | 353 | JF768210 |
| 46 | LcSSR46 | F: AATGCATGGGTTAGGTTCCA  R: AGCTCCCCAAAAGGGTAAAA | (GAT)_4_…(GA)_13_…(GAAT)_2_ | 60 | 388 | JF768211 |
| 47 | LcSSR47 | F: TGAGGGAGATTTCCAAGAAAGA  R: AAACTTGATGAAGATCAGAGTTGTTC | (GTTT)_2_…(CT)_8_ | 60 | 221 | JF768212 |
| 48 | LcSSR48 | F: CTACGCTAGCCTTTTCAACG  R: ACAACCGTGGTCACTCAAGA | (CT)_7_…(CA)_5_…(GAA)_5_ | 59 | 381 | JF768213 |
| 49 | LcSSR49 | F; TTCAAAAGATAAGATGTTGAGATGC  R: TTTGGGAGTATTTCAAGGATGA | (GA)_9_…(AT)_4_ | 58 | 259 | JF768214 |
| 50 | LcSSR50 | F: TCCTCCATAGGAGGTGTTGA  R: TCCAACTTCCTAACTAACTGAATAACA | (GA)_8_…(GA)_5_…(AT)_4_ | 58 | 295 | JF768215 |
| 51 | LcSSR51 | F: TGGGAGATTTCCAAGACTGG  R: TCAGTAAAAGAAGGTACAAGAGTTGC | (CT)_7_ | 60 | 246 | JF768216 |
| 52 | LcSSR52 | F: TGGGAGATTTCCAAGACACA  R: CAAGAGTTGCTTCAAAAAGCTG | (CT)_8_tt(CT)_14_ | 59 | 290 | JF768217 |
| 53 | LcSSR53 | F: TTGGGAGTTTTCCAAGACAGA  R: TTGATGTAGATCAGAGTTGTTCTTGT | (TA)_4_…(GTTT)_2_…(CT)_8_ | 59 | 240 | JF768218 |
| 54 | LcSSR54 | F: GCTTGTTTGAGTTGTCCTTTGTC  R: GGGAAACACCTCAGCAAAAT | (GTAT)_2_…(GAA)_6_ | 60 | 324 | JF768219 |
| 55 | LcSSR55 | F: GCACCGAAAAGGAAAGTTTG  R: AAATAAATCCTCTTGCCCCTTT | (GA)_9_ | 59 | 338 | JF768220 |
| 56 | LcSSR56 | F: TGCTTCAAAAAGATATATCACAACAG  R: TAAAGGCGTTTACCATGCTG | (CTT)_3_…(GA)_8_ | 58 | 348 | JF768221 |
| 57 | LcSSR57 | F: TCACAACAGTGATGTTTTACACAA  R: TTGTGGGAGATTCCAAGACA | (CTT)_3_…(GA)_7_…(GTT)_3_…(GTT)_3_ | 59 | 278 | JF768222 |
| 58 | LcSSR58 | F: TCACAACCGTGGTCTCTCAA  R: TGGGAGTTTTCCAAGACTGAA | (GA)_12_…(GTT)_3_ | 60 | 331 | JF768223 |
| 59 | LcSSR59 | F: CTTCAAATGATAAGTTGATAAGATGC  R: TTTGGGGTATTTCAAGGATGA | (GA)_19_ | 59 | 272 | JF768224 |
| 60 | LcSSR60 | F: TTCAAAAAGCTTTATCACAACAGTG  R: TGGATCAAGTGGTATATTTGAAGAA | (AAT)_4_…(GTGTT)_2_ | 59 | 236 | JF768225 |
| 61 | LcSSR61 | F: CCGATCAAATCCAGATACGG  R: TGCTGTATTTATTGTTGTATTCATGG | (CAA)_4_ | 60 | 326 | JF768226 |
| 62 | LcSSR62 | F: AGTAAAGTGATAATGGGCCAAA  R: ATCCAATCCCTCCTTGATCC | (GA)_16_…(CT)_4_ | 60 | 281 | JF768227 |
| 63 | LcSSR63 | F: CACACTCACTAATCCACATACATCC  R: GTGACCGGTGAAAAACTCGT | (GA)_4_aa(GA)_3_…(CT)_7_…(CTT)_4_…(AT)_3_ac(AT)_2_…  (CCA)_5_…(CAA)_4_ | 60 | 486 | JF768228 |
| 64 | LcSSR64 | F: CCGCGAAGCATTCAAATAAC  R: TTTTCACTCCTCTCCCGTTC | (GA)_3_…(GA)_5_ggg(GA)_7_ | 60 | 283 | JF768229 |
| 65 | LcSSR65 | F: AAATGCTTCTAGAAAAGCTGTAATCA  R: TGGAACAAGTGGTATATTTGGAGA | (GA)_7_ | 59 | 277 | JF768230 |
| 66 | LcSSR66 | F: TCCTTGCTGAATTGCATGTT  R: GAGCCACTGGTCCATTCATT | (GA)_13_ | 59 | 266 | JF768231 |
| 67 | LcSSR67 | F: ATTGTGGGAGTTTTCCAAGG  R: GCCCTAGTCTCTCAAGGAACAA | (CT)_11_…(CA)_7_ | 59 | 397 | JF768232 |
| 68 | LcSSR68 | F: TCTCGAGAATCCTGACTAGCAA  R: TGGAACAAGTGGTATATTGGAGAA | (GT)_4_…(GAT)_3_…(CTT)_4_…(GA)_8_ | 59 | 380 | JF768233 |
| 69 | LcSSR69 | F: TTGCCAAATGATAACGGTGA  R: TTTCCTCTTAGCATTCTTTACGC | (GAA)_7_ | 59 | 230 | JF768234 |
| 70 | LcSSR70 | F: CCGTAAAGTATGACCCATTTGA  R: ACCTTCAATCTCAGTGCTACCC | (CT)_17_…(CA)_17_ | 59 | 300 | JF768235 |
| 71 | LcSSR71 | F: CCTTTAATAACATTCTCATTTGTGG  R: ACTTTCAAAGCCACCTTCAA | (GA)_13_…(AT)_4_…(AT)_4_ | 58 | 300 | JF768236 |
| 72 | LcSSR72 | F: GGATTTTTAGCTTAAAGGGAACAC  R: TTAAAACCTTTGCTCCGGTA | (CT)_16_ | 58 | 250 | JF768237 |
| 73 | LcSSR73 | F: AAACTTTGATGACGATCAGAATG  R: TGAGGGAGATTTCCAAGACAA | (CTT)_3_…(GA)_17_ | 59 | 267 | JF768238 |
| 74 | LcSSR74 | F: TTGTGAAGGTGGAAGCAACC  R: GCATGGCTTCTTCTTGGCTA | (GAA)_8_ | 61 | 246 | JF768239 |
| 75 | LcSSR75 | F: CAAATTGAAGAAAGAAAGTGACGA  R: TTGCTTCAAAAAGCTATATCACC | (CT)_8_…(GAA)_3_ | 59 | 233 | JF768240 |
| 76 | LcSSR76 | F: CCAAAAGAAGAAGATTTGGTTAAG  R: CTAGGAACAAGATCGATGGTAAA | (GA)_18_ | 57 | 250 | JF768241 |
| 77 | LcSSR77 | F: AACCCAATGTCTCTGTTGTTTTT  R: AGATCTTAGTAAACATGAGTTTTGAGG | (GT)_4_…(GA)_15_ | 58 | 308 | JF768242 |
| 78 | LcSSR78 | F: CAGTTTACAAGAAACCAATAATGTG  R: TGTGGGAGATTTCCAAGACTG | (CTT)_3_…(GA)_8_ | 60 | 291 | JF768243 |
| 79 | LcSSR79 | F: TGCCATGTCAGCATAACATTT  R: AGGTTGGGCCTAACTCATAATTG | (AT)_4_…(CT)_7_ | 60 | 280 | JF768244 |
| 80 | LcSSR80 | F: CACAAGTTAAGGGCAATGACA  R: AGTGCTCTTGTGGCTTGTGA | (GA)_7_…(AT)_4_ | 59 | 246 | JF768245 |
| 81 | LcSSR81 | F: GCCCTAGTCTCTCAAGGAACAA  R: TCAAGAAAGAGAAAAACAACACAC | (CTT)_4_…(GA)_10_ | 59 | 300 | JF768246 |
| 82 | LcSSR82 | F: AAGGGATGTGTTGGGTGAAA  R: GGCAATGATGAAAATGATGG | (GA)_5_gg(GA)_4_…(GAA)_5_ | 60 | 388 | JF768247 |
| 83 | LcSSR83 | F: TGGTTCACCAAAATGTATAATGC  R: CCTTCAATCTTAGGTAACTGCTACG | (CT)_12_(CA)_14_ | 59 | 229 | JF768248 |
| 84 | LcSSR84 | F: CATCTGTTGGATCAAAGCATTAAC  R: TGGGGTTACACCCTCACTAAA | (GA)_11_ | 59 | 361 | JF768249 |
| 85 | LcSSR85 | F: GTCAGCACACGATTGGACAT  R: CAAGAGTTGCTTCAAAAAGCTG | (CT)_8_ | 59 | 282 | JF768250 |
| 86 | LcSSR86 | F: TCCCAGATATTCTCCTTTGCTC  R: GGCATGGGTTTTTGTTTGAA | (CT)_18_…(CT)_4_ | 60 | 372 | JF768251 |
| 87 | LcSSR87 | F: CCCAATACTCTTCCCCTTGTT  R: CCCTCTCAAATCATGGCATC | (GA)_6_…(GA)_9_ | 60 | 400 | JF768252 |
| 88 | LcSSR88 | F: TGTTTTCCTTCATGCTGGATT  R: GGCACCAGTTTGGTTTCTTT | (GA)_29_ | 59 | 284 | JF768253 |
| 89 | LcSSR89 | F: TCCTCCATAGGAGGTGTTGA  R: TTCACGTCACCTGCAAAAAG | (GA)_8_…(GA)_4_…(GTTA)_2_ | 59 | 389 | JF768254 |
| 90 | LcSSR90 | F: TCAACGGCTCCTTTGTTAGAA  R: TGCTTCAAAAAGATGTATCACAAAC | (CT)_8_ | 59 | 299 | JF768255 |
| 91 | LcSSR91 | F: AATCGTTCTCATAGGGAAAAGTTC  R: CGACTTATACTTAGAAATGAAGGGAGT | (CT)_11_ | 59 | 298 | JF768256 |
| 92 | LcSSR92 | F: GCCTTTTCAACGGCTACTTT  R: GCTTCAAAAAGCTTTATCACAACA | (CT)_7_…(GAA)_3_…(ATT)_3_ | 59 | 295 | JF768257 |
| 93 | LcSSR93 | F: GGGAAAAGTTCCCTAATCATTTT  R: ACATTTAGAAATGAAGGGAGCTAA | (CT)_10_ | 58 | 280 | JF768258 |
| 94 | LcSSR94 | F: TGATTAACTTTGATGAAGTGCAGA  R: TCCATGACTTTTCAACGGTTAC | (GA)_20_ | 59 | 310 | JF768259 |
| 95 | LcSSR95 | F: ACACAACCGTGGTCTCTCAA  R: GCCTTTTCAACGGCTACTTTC | (CTT)_3_…(GA)_6_ | 60 | 361 | JF768260 |
| 96 | LcSSR96 | F: CATATGAGTCGTATTTGCAGTGG  R: GCTCCTTGGCCAAACTATCA | (GA)_8_…(ATTT)_2_…(GTT)_3_ | 60 | 366 | JF768261 |
| 97 | LcSSR97 | F: GCGCAAGATTGGACATAAAGAT  R: TCTTTCTCTTGAGTATGAACGTG | (CT)_9_ | 60 | 284 | JF768262 |
| 98 | LcSSR98 | F: GGATGGATTTTCATCTTCCTTC  R: GGTTCTACCTGCTCTAACCAATG | (GA)_7_ | 59 | 297 | JF768263 |
| 99 | LcSSR99 | F: GGAGTTGTCCAATATCGAATACA  R: ATTGCAAAACCCCCTGGATTAC | (CT)_8_ | 63 | 295 | JF768264 |
| 100 | LcSSR100 | F: GGGGAGTTTTCCAAGACTGAAT  R: TGGTTTACAGGGTACACAGAGTG | (CT)_7_ | 60 | 300 | JF768265 |
| 101 | LcSSR101 | F: CTGAATTAAGGGATGGTGTTACT  R: TCGACACATTGTTTCATGTGG | (CT)_5_CA(CT)_4_ | 60 | 250 | JF768266 |
| 102 | LcSSR102 | F: AGGCGGAAGGAATGGTAAAT  R: CACAATTAGTGAGTGTGTGATTTGA | (CTAT)_17_(CT)_5_AT(CT)_20_ | 59 | 358 | JF768267 |
| 103 | LcSSR103 | F: CCTGCTTGAGAATCTGACACAC  R: TTTCTAGCCTTCAACGTCCTTT | (CTT)_5_…(GA)_12_ | 59 | 391 | JF768268 |
| 104 | LcSSR104 | F: ATCCTGACTAAGCCCCATCTCT  R: TGGGAGTTTTCCAAGACAGAAT | (CT)_5_…(AAT)_3_…(GA)_6_…(AATAT)_2_ | 60 | 379 | JF768269 |
| 105 | LcSSR105 | F: GGAGTTTTCCCCAACACAAATA  R: GCATGGGCTTCAAAATGAAAT | (CTAT)_2_…(CT)_4_ | 61 | 272 | JF768270 |
| 106 | LcSSR106 | F: TAGGGCAAAAGGGCACAA  R: ACTCAGAAATGAGTCCCAGAGC | (GA)_10_ | 60 | 398 | JF768271 |
| 107 | LcSSR107 | F: GACCACCCTCTACTTTTCAACG  R: GATCAAACTGAGATGCAATGGA | (AATAT)_2_…(CT)_5_…(GA)_5_ | 60 | 491 | JF768272 |
| 108 | LcSSR108 | F: CACTTCCTTGATAGTTCTTTTCTCA  R: AATTAGCAGACCGCTTTTCC | (AT)_5_…(AT)_4_…(CT)_32_ | 58 | 399 | JF768273 |
| 109 | LcSSR109 | F: ATTGAGGGAGATTTCCAAGACA  R: TGACACTAGCATACAGCCGAGA | (CAA)_3_…(CT)_15_ | 60 | 374 | JF768274 |
| 110 | LcSSR110 | F: TCTCCACTTCTCCCTTTCTTTG  R: CTATAGCCGGATCAAAGTCCAC | (GA)_17_ | 59 | 309 | JF768275 |
| 111 | LcSSR111 | F: GGTGTTTACCATGCTGCTAGTC  R: GGATACTTTTGTGTACGGGTGA | (CT)_7_ | 58 | 394 | JF768276 |
| 112 | LcSSR112 | F: CGGAGGAGCTTAATCCATAGAA  R: TTAACAGCTTTTCCAATCTCAGC | (GA)_8_ | 59 | 350 | JF768277 |
| 113 | LcSSR113 | F: CCCTATTCGTGAACCCTAACAC  R: CGCGACTCAGATAATGACAAAG | (GA)_7_ | 59 | 380 | JF768278 |
| 114 | LcSSR114 | F: GAGAGTCTGATTTGGTTTGTCTT  R: CCAAAAGGAAAGTGATGTTCGT | (GA)_28_ | 60 | 376 | JF768279 |
| 115 | LcSSR115 | F: CTGACTCACAACCATGGTCACT  R: CCACTCTAGCCTTTTCACGACT | (CTT)_4_…(GA)_11_…(AAT)_3_…(ATT)_4_ | 60 | 377 | JF768280 |
| 116 | LcSSR116 | F: CACATAGACGAATTCCCTAATCA  R: CAGAATGCTTCGTAAGTCAAGG | (CT)_6_ | 59 | 359 | JF768281 |
| 117 | LcSSR117 | F: CAATGGAACCACTTAAACAACC  R: CTTAGCATTTTCACGGCTACTG | (CTT)_4_…(GA)_7_…(CTT)_3_…(AT)_4_ | 59 | 470 | JF768282 |
| 118 | LcSSR118 | F: TCCAAGGTTAATCTTCTTCCTC  R: GTGCAGTGAGTCAGCGTAAGAT | (GA)_6_ | 59 | 352 | JF768283 |
| 119 | LcSSR119 | F: ACCTAGTCTCTCAAGGAACAACT  R: CTTTTCAACGGTTACTTCTATGG | (GA)_8_…(AAT)_2_ | 57 | 400 | JF768284 |
| 120 | LcSSR120 | F: CCCGAGAAATCTGACTAGCAAC  R: TACTCAAGCTATGCATCCAACG | (GA)_12_ | 60 | 452 | JF768285 |
| 121 | LcSSR121 | F: TAGTGTGAGTACCGGGGTGGAATA  R: TCTCACATTTAGCTTGCTCCAA | (GATA)_12_…(GA)_18_ | 60 | 367 | JF768286 |
| 122 | LcSSR122 | F: CCATGCTGCTAGCCTACTACAA  R: TGTAATGTGATTATGGGGGAGA | (AATAT)_2_…(CAA)_3_…(CT)_17_ | 59 | 288 | JF768287 |
| 123 | LcSSR123 | F: GCAAATTTCCAAAACACATCC  R: TGGTGAATTACTAGTTCGGCTTT | (CT)_5_ | 59 | 242 | JF768288 |
| 124 | LcSSR124 | F: CATAACGGTGTTTACCATGCTG  R: CAGCAATATGAACCGAATGAGA | (GA)_7_…(CT)_4_…(CT)_4_…(GAA)_3_ | 60 | 459 | JF768289 |
| 125 | LcSSR125 | F: CTTCCGGAAAAATCCTTGGT  R; AACATGGGACGCTATCTGTTTT | (GAA)_3_…(CT)_4_ | 60 | 386 | JF768290 |
| 126 | LcSSR126 | F: CACCTACGTCCTGTGCATTTT  R: TACGCGTGGGCTTACATATCTC | (CTT)_4_…(GAT)_3_…(GA)_4_t(GA)_5_ | 61 | 497 | JF768291 |
| 127 | LcSSR127 | F: AAAGCCCTAGTCTCTCAAGGAA  R: TGGGAGATTGCAAGACTGAATA | (CTT)_3_…(GA)_11_ | 59 | 361 | JF768292 |
| 128 | LcSSR128 | F: GATGGAGAAACAGGGTATCCAC  R: TTGTTTTCGAAGGAATGTTGG | (CT)_4_…(CT)_4_ | 59 | 297 | JF768293 |
| 129 | LcSSR133 | F: CGTTAAGTTGAGTCGAATTTGTG  R: GCAACTTTCAACATTGCCATT | (GTT)_3_ | 60 | 245 | JF768298 |
| 130 | LcSSR135 | F: AACCTGTTTGTTTCTCCATCCT  R: GGTGAAGTCTGTGATATGTTTCT | (CT)_3_tt(CT)_3_…(CACT)_2_…(CT)_9_ | 59 | 300 | JF768300 |
| 131 | LcSSR139 | F: CAACAAGCTGTATCACAACAGTG  R: TTTTGGGAGATTTCCAAGACAC | (CTT)_8_…(GA)_7_…(GT)_4_ | 60 | 300 | JF768304 |
| 132 | LcSSR141 | F: GGAAAAGTGGTATCTTGCATTG  R: TGAGTACGTGTGGATTAACAGTGA | (CT)_3_cc(CT)_4_ | 59 | 279 | JF768306 |
| 133 | LcSSR144 | F: CATCTAGAATACCACCCCTATTATC  R: TGATACCTCGTTTTCGAGTC | (ATT)_3_…(GT)_14_…(GA)_5_ | 56 | 300 | JF768309 |
| 134 | LcSSR145 | F: AACCGACGACTGTAGAATTATC  R: GGATCAAGTGTATTTTCGGACA | (GA)_8_ | 58 | 399 | JF768310 |
| 135 | LcSSR147 | F: GGTAACAGTGGAAAAATGGAA  R: AGAATGCTTGCCTATAAATGG | (CT)_4_…(CT)_11_ | 56 | 300 | JF768312 |
| 136 | LcSSR149 | F: CAGTCTTTTCACCCCTCTTTTC  R: GGGTTTAATCTCCCTCTTGCTT | (CT)_6_ | 59 | 393 | JF768314 |
| 137 | LcSSR150 | F: GGCTGATTTGGTGAAAGAAGAA  R: CAATATTATTCAGGGGCTTTGG | (CA)_4_…(CT)_19_…(CT)_7_ | 60 | 437 | JF768315 |
| 138 | LcSSR151 | F: AAAGTCAAAACTCTCCATGTTGC  R: TCACTAAGTGTGGAGTAACACAGAA | (GAAA)_3_…(CT)_17_ | 59 | 398 | JF768316 |
| 139 | LcSSR154 | F: CTCGTTTATACCGATAGAAGAATCC  R: ATTTAAAAGGACCGCTGACTGA | (GA)_7_gg(GA)_4_ | 60 | 338 | JF768319 |
| 140 | LcSSR156 | F: TGGATCAGTGGTATATTTGGATG  R: AAGGGAGCTCTCCCATTATGA | (CT)_7_ | 60 | 297 | JF768321 |
| 141 | LcSSR157 | F: TGGGAGTTTTCCAAGACTGAAT  R: CTTACGCGTGGAGTAACAAAGA | (CT)_11_…(GAAA)_3_ | 59 | 284 | JF768322 |
| 142 | LcSSR158 | F: GACTACGCTAGCCTTTTCAACG  R: TGATTACGCGTGGAGTAACAAG | (CT)_8_ | 60 | 326 | JF768323 |
| 143 | LcSSR159 | F: GACCAATCTAGCCTTTTCAACG  R: GCAAGGACTAACGAGAGTTGCT | (AATAT)_2_…(ATATT)_2_…(CT)_9_ | 60 | 334 | JF768324 |
| 144 | LcSSR160 | F: CAATCCCAAGCCTTCAATTCTA  R: GACTCTTGCTGTCCGCATATTA | (GTT)_3_…(CT)_9_ | 60 | 265 | JF768325 |
| 145 | LcSSR162 | F: CAGAGTGCAAATTGCGTTGTAT  R: GGCAAGACAAAGTAGCTCAGAAA | (GA)_4_…(GA)_4_ | 60 | 351 | JF768327 |
| 146 | LcSSR165 | F: CCTTTTCAACGGCTACTTTGTT  R: AGCTCCACTCAAAGCAAACACT | (CT)_4_ tt(CT)_4_…(GAA)_3_ | 60 | 389 | JF768330 |
| 147 | LcSSR167 | F: CCTTTTCCTTTAGATTGGCATC  R: TCAAGTGAATAATGCAGCAAGC | (CT)_5_…(GA)_3_tc(GA)_5_ca(GA)_5_…(CT)_4_ | 60 | 458 | JF768332 |
| 148 | LcSSR169 | F: ACAACGATGAACCTTTCTGTCC  R: AAACTCAGAAGGGTGCGTGA | (CT)_4_att(CT)_5_…(CT)_4_ | 60 | 229 | JF768334 |
| 149 | LcSSR170 | F: GGGAGATTTCCAAGACTGAATA  R: ACCGTGGTCACTCAAGAATA | (CT)_8_ | 57 | 300 | JF768335 |
| 150 | LcSSR171 | F: CCGGAAATACGATCACTGACAT  R: GTTGTATGGCCCAAAGTCAAAT | (GA)_8_…(GA)_9_ | 61 | 274 | JF768336 |
| 151 | LcSSR173 | F: GGGATATATGCCAACTGAGGAA  R: GCATTGAGTCATCACACGATAA | (CT)_4_gg(CT)_4_ | 60 | 274 | JF768338 |
| 152 | LcSSR174 | F: GGCATTCCATGCTTTTACATTC  R: TTTGATTTCGATCCAACAGAG | (CT)_6_ | 60 | 287 | JF768339 |
| 153 | LcSSR175 | F: TTTGGGAGTTTTCCAAGACAGA  R: GAATTCACTGGCGATTCTCG | (GAA)_3_…(CT)_8_…(GAA)_3_…(CTTT)_2_ | 60 | 300 | JF768340 |
| 154 | LcSSR178 | F: CAAAGATTTGCTTCTAAAAAGCTG  R: TTGCAAGAGATTTCCAAGTATGA | (GA)_8_ | 58 | 277 | JF768343 |
| 155 | LcSSR180 | F: ATTGTCGAGCTGCCTGTATTC  R: CCAAATTGACGACGAAAA | (GA)_4_…(GAAA)_2_…(GA)_16_ | 59 | 500 | JF768345 |
| 156 | LcSSR182 | F: TTTGGACAGAGTATATAAAGGATTC  R: AAACCTGTTTCTTCTCTTGATATG | (CT)_10_ | 55 | 139 | JF768347 |
| 157 | LcSSR183 | F: GACTACGCTAGCCTTTTCAACG  R: AACATATATCGCAAGGTGTGATG | (ATATT)_2_…(CT)_8_ | 60 | 296 | JF768348 |
| 158 | LcSSR185 | F: TTGACCCTTGGCATATGTCTC  R: GAATGAATGCCATGGAAAGAA | (CT)_5_…(CT)_7_…(GTTT)_2_…(AAAT)_2_ | 59 | 298 | JF768350 |
| 159 | LcSSR186 | F: GACAACCGTCTAAGTCTTCTCG  R: TCATATAACATTCAAAATGAAACAA | (GA)_4_aa(GA)_26_ | 58 | 342 | JF768351 |
| 160 | LcSSR187 | F: GCAAAGAAATGTGGTAATGTGC  R: CGAAACTTACACTTCCCAAACC | (GA)_10_aa(GA)_3_…(CTTT)_2_…(GA)_4_…(GA)_4_ | 59 | 396 | JF768352 |
| 161 | LcSSR188 | F: GGTTCTAATTCCCAATTCAAGC  R: GTGTGTTAATGACACGATCAACT | (CTTT)_2_…(CT)_12_ | 59 | 388 | JF768353 |
| 162 | LcSSR190 | F: GCTCTCTAACTGATTAGCCGACA  R: AAGGCAAATATACCCACACACTT | (GA)_15_gc(GT)_3_gc(GT)_5_(GA)_4_ | 60 | 353 | JF768355 |
| 163 | LcSSR191 | F: GTTGCTTCCACGAGCAAAAAT  R: TCTTCTTCCCAACAACAAAGA | (GTTT)_2_…(GA)_5_ | 61 | 387 | JF768356 |
| 164 | LcSSR194 | F: CCAAAGGCAACAATCAAAACA  R: TGGGTTAGATCAAGGGAAAAGA | (GA)_12_…(ATTT)_2_ | 60 | 239 | JF768359 |
| 165 | LcSSR199 | F: GGAGTTTTCCAAGGCTGA  R: TCTCCAAATATTCACTTGATCC | (CT)_5_ | 56 | 275 | JF768364 |
| 166 | LcSSR203 | F: TAGTGTCTCAAGAGTGCCCAGA  R: CACCCACTTGGAAAGGTCAT | (GA)_3_…(ATT)_2_ | 60 | 205 | JF768368 |
| 167 | LcSSR204 | F: TGGGCTTATGACAAAAACGA  R: TGCGTAGCTTTGATGTTTGAA | (CCG)_3_…(CT)_3_cc(CT)_2_ | 59 | 356 | JF768369 |
| 168 | LcSSR205 | F: CATGAATGGAGGAAATCCAA  R: TGATCAGAGCATGACCCACT | (GT)_2_at(GT) | 59 | 238 | JF768370 |
| 169 | LcSSR206 | F: TTCCACCATTGGAACCTAAA  R: GCTTAGAAATCACAAGTTCTCTCTC | (GA)_8_ | 57 | 237 | JF768371 |
| 170 | LcSSR207 | F: TGATTCGGGAGAAGATGAGG  R: ATCATGCAGATTCGCAAGC | (GGAGA)_2_ | 60 | 248 | JF768372 |
| 171 | LcSSR208 | F: CATTTACTTCACAAGCTTTATCACA  R: GGAACAAATTCATCCAAGAGG | (CTT)_3_…(GA)_7_…(GTT)_3_ | 58 | 270 | JF768373 |
| 172 | LcSSR209 | F: CGACAATTGCTGATGGAATG  R: TCGACGGTCTCTTGATTCGT | (GAGAA)_2_ | 60 | 283 | JF768374 |
| 173 | LcSSR213 | F: AGCTTTTCCAACGGCTATTT  R: GGAGAAAGCACTTGGTAACAAAA | (CT)_4_ | 59 | 295 | JF768378 |
| 174 | LcSSR214 | F: AACAATCAGAACAGAAAGGACA  R: AGACTAGCAGCATGGAAAAA | (ATATT)_2_…(GT)_3_ | 56 | 193 | JF768379 |
| 175 | LcSSR215 | F: AGAGTCAGTTGATTGATGTTGTTG  R: CCCTGAGTGCTACGTATCAGAA | (AT)_4_…(ATT)_3_ | 59 | 299 | JF768380 |
| 176 | LcSSR216 | F: AATGACCGTTGGAGTCAATG  R: TTCCATTTGTCCACTGATTAACTT | (AAAT)_4_…(AT)_3_…(GAAT)_2_ | 58 | 373 | JF768381 |
| 177 | LcSSR217 | F: TCGGTTTGCTGGTTCAATAA  R: GCTCAAGCATAGTCGTATACTCAGA | (CT)_5_…(CT)_4_…(CTCTT)_2_ | 59 | 250 | JF768382 |
| 178 | LcSSR219 | F: TCATTCAAAATGAAGCCACCT  R: AATTCCAATGTGCTCTGTCG | (CG)_3_…(GA)_4_ | 59 | 300 | JF768384 |
| 179 | LcSSR223 | F: GACTGTGCACGGATGTAAAAA  R: AGCAATTTCCAAATGGCATC | (GT)_4_…(GA)_4_ | 59 | 227 | JF768388 |
| 180 | LcSSR224 | F: AAAGGTGTTTACCATGCTGCT  R: GCCCGGTCTTACTTTTGATTT | (CT)_8_…(GAA)_4_ | 59 | 495 | JF768389 |
| 181 | LcSSR225 | F: ATATACCGGTGGGATTGAGG  R: GCAACAGGGTGAATGAATGA | (AT)_5_…(AT)_4_…(CTAT)_2_ | 59 | 381 | JF768390 |
| 182 | LcSSR227 | F: ATACGTAACCCTCGGGGAAT  R: CGCACCATCATTCTTCAGTG | (ATT)_3_ | 60 | 474 | JF768392 |
| 183 | LcSSR228 | F: CTCGATTTTGTTTTCTTGCTGA  R: ATGAAATCAAGGCGGAACAA | (GAAA)_2_ | 60 | 192 | JF768393 |
| 184 | LcSSR233 | F: GAACAATATTGCTGCTTTTCACTT  R: CGCCCCCACAATTTACAATA | (AT)_5_ | 60 | 234 | JF768398 |
| 185 | LcSSR236 | F: TTTTCAACGGCTCCTTTGTT  R: AACCGTGGTCACTCAAGACTATT | (AATAT)_2_…(CT)_5_…(GAAA)_2_ | 59 | 363 | JF768401 |
| 186 | LcSSR238 | F: TTTCGAGCAGTTGATTGTGG  R: CAGCCAATGTCCATTTACATCA | (CT)_2_…(CT)_3_…(GGA)_3_ | 60 | 372 | JF768403 |
| 187 | LcSSR241 | F: CGGAGAATTATCATTGGTTGG  R: TGCTAGTTTTCGAAATGATGCT | (AAATC)_2_ | 59 | 248 | JF768406 |
| 188 | LcSSR243 | F: TTTATGGCACGTGTTTACCG  R: CGGGTTATGTCCACAAACAA | (GGAAA)_2_…(GA)_4_ | 59 | 390 | JF768408 |
| 189 | LcSSR246 | F: GGAGAGATTTTTACTCAACCCACA  R: TGAGGATGATGATGAAACTCCA | (GACA)3 | 60 | 238 | JF768411 |
| 190 | LcSSR247 | F: TTTTCTTCGCCTTCTTATGC  R: AATTGCATGCAGGGTATCAG | (AT)_2_a(AT)_2_…(CT)_3_ | 58 | 369 | JF768412 |
| 191 | LcSSR250 | F: GAAGAATCCGGATGAATCACA  R: CCGGTGGATACAAAAGATTG | (GT)_4_ | 59 | 298 | JF768415 |
| 192 | LcSSR252 | F: TCCATGCTGGGATTCTAATTG  R: CACATCAGCGATGTTTTACTCA | (CT)_5_…(CT)_4_ | 59 | 304 | JF768417 |
| 193 | LcSSR253 | F: TGCAACACACAGTCATTTCCT  R: TGGGGATACCTAAGCATTTCA | (AT)_5_ | 59 | 357 | JF768418 |
| 194 | LcSSR255 | F: TGTTGCTATGTTATAAGCTGTTGA  R: AAAACTTCAATCTCGATTACCTG | (AT)_2_g(AT)_3_ | 56 | 377 | JF768420 |
| 195 | LcSSR256 | F: TGAAATTTTTGGGGCACAAT  R: TTCTTCGTTCTCTTTCTCCCTCT | (GAAA)_2_…(GA)_5_ | 60 | 397 | JF768421 |
| 196 | LcSSR257 | F: AGGCATAGACCGTTTAGTGAACA  R: GAGCTTTTTACCGTACATCCAAA | (CT)_4_ | 60 | 287 | JF768422 |
| 197 | LcSSR259 | F: TGACCGTTCAGTAGCAATTCA  R: CCATTTTTGACCTAAGTATCACGA | (CA)_4_…(CA)_4_ | 59 | 297 | JF768424 |
| 198 | LcSSR260 | F: TCACCACCTTTCTGTGCTTTT  R: TATTTATTCCGCTGCTGCAA | (GAAT)_2_…(GA)_4_…(GA)_4_…(GA)_4_…(GA)_4_…(GA)_4_ | 59 | 398 | JF768425 |
| 199 | LcSSR262 | F: ATCTGAAGGGTGGACAATGA  R: GTGGACGAACCAATCAAAGT | (CAA)_3_…(CT)_4_…(CAT)_3_ | 57 | 378 | JF768427 |
| 200 | LcSSR263 | F: TCAGCATGTCATCTACATCGTCT  R: TCACCGGATGCATTATCAAG | (CT)_4_ | 59 | 281 | JF768428 |
| 201 | LcSSR265 | F: CTTTTATACCGATAGAAGAATCCA  R: AAATACAAAATTCGTTCTCATCC | (CATA)_2_…(GA)_19_ | 56 | 394 | JF768430 |
| 202 | LcSSR266 | F: TCCTGGCCTACAGGTTCAAA  R: TTGCAATCAAAAGAACAAAGTGA | (CT)_4_…(CTTT)_2_…(ATTT)_2_ | 60 | 247 | JF768431 |
| 203 | LcSSR267 | F: TCTATTCTTTCCTTTCTACATCTCG  R: GCTTAGGTTAAATCAACCAGAGTAA | (CT)_2_gt(CT)_3_ | 57 | 250 | JF768432 |
| 204 | LcSSR269 | F: TCAAGGTCAATGCCACTGTAA  R: TTCCTTAACCTAGCCGGAAA | (GA)_5_…(GA)_4_…(GA)_4_ | 59 | 367 | JF768434 |
| 205 | LcSSR271 | F: TCGTTATTTTTCCCTTCCTCTT  R: TTTTGACCAAGGCTTGTTTT | (CA)_4_…(ATTT)_2_ | 58 | 242 | JF768436 |
| 206 | LcSSR273 | F: GGAAGAATAACTCACTCAAGAGGAA  R: CCTTTTCTGAACCATCGTTTG | (GA)_4_…(GAGT)_2_…(GA)_5_…(GGA)_5_…(GAAT)_2_ | 59 | 275 | JF768438 |
| 207 | LcSSR274 | F: GATTCATCAATCCCGTCCAT  R: TCCCCTAAGCCAAACACAAC | (GGGAAA)_2_…(CT)_2_at(CT)_3_ | 59 | 465 | JF768439 |
| 208 | LcSSR275 | F: CGAGGTATGATACGTTGATGTGA  R: CTGTTGTGAATGAAAAACTTGAGAG | (CT)_9_atctat(CT)_8_ | 59 | 300 | JF768440 |
| 209 | LcSSR279 | F: TGGGAGATTTCCAAGACAAAA  R: TCTCTCAAGGAACAAATGCAA | (GAAA)_3_ | 59 | 385 | JF768444 |
| 210 | LcSSR280 | F: AAGGTTGATTGAGTATCTCGGTTC  R: TGCACATGCACACACTAAACA | (CT)_4_..(CT)_6_ | 59 | 300 | JF768445 |
| 211 | LcSSR281 | F: GCAACTCTAGTCTCTCAAGGAAAA  R: CACTAGTGACTCTCTTGGCTTACG | (GA)_6_a(GA)_4_ | 59 | 385 | JF768446 |
| 212 | LcSSR282 | F: AGAAATCTGACTCACGCCTGT  R: AGAGTACGTTTACACCATGCAG | (CTT)_4_…(GA)_8_…(CTT)4 | 58 | 473 | JF768447 |
| 213 | LcSSR284 | F: GCTTCAAAAAGCTTTATCACAACAG  R: GTGGCGACTCTTGCTTCATT | (CTT)_5_…(GA)_7_ | 60 | 334 | JF768449 |
| 214 | LcSSR287 | F: TGCAGTGTGTCAGCGTAAGA  R: GACTTGATGAAGATCAGGGTGTT | (CT)_8_…(GAA)_3_ | 59 | 359 | JF768452 |
| 215 | LcSSR288 | F: GGAGGAATCTTCAAAGCCATT  R: AGGTGGCTGTGTTGGTGTG | (ATT)_3_…(CT)_4_..(CT)_7_ | 60 | 300 | JF768453 |
| 216 | LcSSR289 | F: TCATACCAGCATAATGGAGTCCT  R: CGATCGGCTTTTGAATGAGT | (CA)_6_ta(GA)_15_ | 60 | 399 | JF768454 |
| 217 | LcSSR292 | F: CAATGTTTGTGTATGAGATTGCTTC  R: TGAATGGGAGAGTTTCACAGG | (AAAT)_2_…(GA)_4_a(GA)_6_ | 60 | 388 | JF768457 |
| 218 | LcSSR293 | F: CATCCCCTCTTGTGCTTGAG  R: CAACATCTGTCATACCAGAATTTCA | (GAT)_6_…(CT)_17_..(CT)_4_ | 60 | 393 | JF768458 |
| 219 | LcSSR295 | F: GCCCTAGTCTCTCAAGGAACAA  R: TGACTGTGTCAGGCAGATTGA | (CTT)_4_…(GA)_12_…(GT)_4_…(AT)_4_ | 60 | 482 | JF768460 |
| 220 | LcSSR296 | F: TGGAAGTTTAGCTCGCGTAAT  R: TCCAAGGTCGTGAGTTCAAA | (CT)_4_..(CT)_5_..(CT)_21_ | 59 | 494 | JF768461 |
| 221 | LcSSR297 | F: TGTTCATGAATTGCCTCACTTT  R: AACGGACATTCGGACAAGAG | (AT)_5_ | 60 | 240 | JF768462 |
| 222 | LcSSR299 | F: CCTTTTAAAGTATCGCAAACGA  R: AAAACTTTGGAGAAAGAGAGTGTG | (CT)_15_(CA)_8_ | 58 | 247 | JF768464 |
| 223 | LcSSR302 | F: TTTGGTTGGTTGAAGTTTGG  R: TGCCTAATCCAAAGGGAGTG | (GAAA)_3_ | 60 | 244 | JF768467 |
| 224 | LcSSR304 | F: CTCAAGGAACAAACCGCAAT  R: CTTTTCAGCGAACTCCTGGT | (CAAA)_2_…(CTT)_3_…(GA)_8_…(AT)_4_ | 60 | 397 | JF768469 |
| 225 | LcSSR306 | F: GGTTGCTTCAAAAAGCTATATCAC  R: AGCGCGAGATTGGACTTAAA | (AAT)_3_…(GT)_6_(GA)_14_ | 59 | 393 | JF768471 |
| 226 | LcSSR308 | F: AAAGTTAATTTCTCCATGCTGCT  R: TTCTTCTTGATATGAACGTGAGAGA | (CT)_16_ | 59 | 292 | JF768473 |
| 227 | LcSSR309 | F: TCGTGAACATCGTTAACATAACC  R: TTCCTAAATTCTCTCATTTCTCACC | (GA)_4_..(GA)_4_..(GA)_4_ | 59 | 343 | JF768474 |
| 228 | LcSSR310 | F: TTCCCCAAGTGCTAAAGTGC  R: TTTTGGGAGTTTTCCAAGACA | (CTT)_4_…(GA)_10_ | 60 | 373 | JF768475 |
| 229 | LcSSR312 | F: GCAAAGAGAGTTTCCCCAATC  R: ACGCGTGTTCCTCACATAGA | (GTT)_3_…(GA)_6_ | 60 | 397 | JF768477 |
| 230 | LcSSR313 | F: ATGTTCGGTTGCAAATTGTT  R: TCAACAATTCGACAAGTTCTACG | (AATT)_2_…(GTT)_3_ | 59 | 379 | JF768478 |
| 231 | LcSSR314 | F: CTGTGGTAGACTCTGATGCATTG  R: GCATGTTTCCTTGAAAATTGG | (ATTT)_2_…(GA)_22_…(CCT)_3_ | 59 | 397 | JF768479 |
| 232 | LcSSR316 | F: AGAAGCCACGCAATTGAAAG  R: GTGATGAAATGCCCCAAAAG | (GA)_16_ | 60 | 265 | JF768481 |
| 233 | LcSSR318 | F: AGAGCTTACTCACGTACCCTCA  R: CCTGTGAAAAATACAGCCAATG | (CA)_4_ | 59 | 384 | JF768483 |
| 234 | LcSSR319 | F: ATGCATTATGTCAGCGCAAG  R: TTGATGAAGATCAGAGTGTTCTTGT | (GAAA)_2_…(CT)_24_ | 59 | 375 | JF768484 |
| 235 | LcSSR321 | F: CGGCACCCTTTACAAAACAA  R: TTACCGCCTTCGAGTTCTACA | (GA)_23_ | 60 | 386 | JF768486 |
| 236 | LcSSR322 | F: TCCAAACCCAAACCAAGAGA  R: CCAAAACTCACGACCCAAAT | (ATT)_3_ | 60 | 220 | JF768487 |
| 237 | LcSSR324 | F: TGCTATTGATATATGGGTTTTGA  R: ACCATCCCCAACTTTTTGAC | (GA)_4_ | 58 | 374 | JF768489 |
| 238 | LcSSR325 | F: TGCTTACCACGGAACTCTATGTT  R: AACCACAGTCGTACGTCGTTAAT | (CT)_4_ | 60 | 237 | JF768490 |
| 239 | LcSSR327 | F: TAAAGCCCTAGTCTCTCAAGGAA  R: TTCAACGATCTTGTAGAAGAAGAG | (CTT)_4_…(GA)_10_…(AT)_4_ | 58 | 386 | JF768492 |
| 240 | LcSSR329 | F: CTTATCACTGAAATATGAAGACTGG  R: CCCCGATTGTAACACTAAACT | (CT)_5_tt(CT)_5_gc(CT)_19_…(CTT)_3_ | 56 | 400 | JF768494 |
| 241 | LcSSR330 | F: ATCACCCGCAACCAAAATAA  R: GGAAAATCCTGAGGTGAGAAAA | (CT)_8_ | 60 | 481 | JF768495 |
| 242 | LcSSR331 | F: CCAGACATTAAATCCAACACAATC  R: CTCGCACAAAATAAACAAAAGG | (CTT)_6_ | 59 | 224 | JF768496 |
| 243 | LcSSR332 | F: CAAGGATTTGCTTCAACAGC  R: CATGCAGATTGTCAGCGTAA | (CTT)_3_…(GA)_9_ | 58 | 366 | JF768497 |
| 244 | LcSSR334 | F: CAAGACTACGCTAGCCTTTTCAA  R: GAGTTGCTTCAAAAAGTTGTATCAC | (CT)_8_…(GAAA)_3_…(GAA)_3_ | 60 | 300 | JF768499 |
| 245 | LcSSR335 | F: AAAGGTGTTTACCATGCTGCT  R: TTGTAAGAGCGATATCACATCAGAG | (CT)_20_…(ATT)_3_ | 59 | 383 | JF768500 |
| 246 | LcSSR336 | F: TCCAGCCTCTTGATTCTCTT  R: TTGTAGTAGATTAGCAGCACTGAAA | (GT)_6_ | 57 | 233 | JF768501 |
| 247 | LcSSR338 | F: TGTCTGCACAAAATTGGACAT  R: ACATTACTGATTGTGCAGCTACG | (CT)_8_ | 59 | 395 | JF768503 |
| 248 | LcSSR339 | F: CAAAAGATCCCGAACGAAAA  R: TCACATCACCTGCAAAAAGC | (GA)_8_..(GA)_5_…(AT)_4_ | 60 | 497 | JF768504 |
| 249 | LcSSR341 | F: CGTATCCGATTTTCTCTCCAA  R: GTTTGGTTTAGAGGATGAAGC | (CT)_4_ | 59 | 244 | JF768506 |
| 250 | LcSSR342 | F: TCCTTAAGATTTTCTTCCCTTGAA  R: CTGGCCTGATCCCTTACAAA | (GAA)_4_ | 60 | 355 | JF768507 |
| 251 | LcSSR345 | F: GCCTTTTCAACGACTACTTTGTT  R: TGCTTCTAAAAGCGTTATCACA | (CT)_16_ | 58 | 300 | JF768510 |
| 252 | LcSSR346 | F: TCATGCTTGAAACTTTGTAGACG  R: GCTGATTGGATAGTAACAGACGA | (GT)_5_ | 59 | 299 | JF768511 |
| 253 | LcSSR347 | F: GGTGTACTAAGAAATGTTTGCTTCC  R: AACGGGTTTAACGCATCAAT | (CTT)_4_…(GA)_5_…(CTTT)_2_…(AAT)_3_ | 59 | 325 | JF768512 |
| 254 | LcSSR349 | F: CAAAGATTGGCATCTAAAAGCTG  R: ATTTTTCGAGTGGTCCCTTG | (GA)_6_ | 60 | 307 | JF768514 |
| 255 | LcSSR350 | F: TGTTTTCCGTGCCACTAATC  R: TGAGTTGGTCTTCTCCTACTCTCAA | (GAAA)_2_…(CT)_4_ | 60 | 274 | JF768515 |
| 256 | LcSSR352 | F: TGAACATGCGAGTGTTCTTG  R: TCAACGGTTATCTCTTGTCGAA | (GA)_8_ | 59 | 278 | JF768517 |
| 257 | LcSSR353 | F: TTTTTGGGTGGGTTCTGTCT  R: AGGCATTTGTTTCGGAAGAG | (CT)_14_ | 59 | 340 | JF768518 |
| 258 | LcSSR354 | F: AACAAACAAACATTTTTCAGTTGG  R: ATCTGAAAGCCTTAGAGTGCAT | (CT)_12_ | 59 | 280 | JF768519 |
| 259 | LcSSR356 | F; TCTCGTAAGTGTTGGTGTAGTTGA  R: CCAGAGAGAATTTACCTTGCTCA | (AAAT)_2_…(GT)_4_ | 59 | 298 | JF768521 |
| 260 | LcSSR358 | F: TGGGAGTTTTCCAAGACTGAA  R: TTGCAAGAAATAAGTCACAAGGTT | (CT)_7_ | 59 | 300 | JF768523 |
| 261 | LcSSR359 | F: GAACCGTGGATTTTCTACATGA  R: TGACCAAATTGTGATTCTCCA | (GA)_4_ta(GA)_17_ | 58 | 393 | JF768524 |
| 262 | LcSSR360 | F: TGGGAGTTTTATGGACGGAAT  R: AACCTGATTGAAGCCCATGT | (GAA)_4_ | 60 | 380 | JF768525 |
| 263 | LcSSR361 | F: TCTTAAATAAGCAGTTTCACAACAG  R: TGCCAAGACAGAAACCAACTT | (GA)_8_(AT)_4_…(GTT)_3_…(AT)_5_ | 59 | 397 | JF768526 |
| 264 | LcSSR362 | F: AGTTTTCCACCACGGACAAT  R: TTCCAAGAGTGTTTTCACGAG | (CT)_4_at(CT)_6_…(GAA)_3_ | 59 | 265 | JF768527 |
| 265 | LcSSR364 | F: CGATACGCTTAAGGGAATTGTA  R: CCAAGCGGTAACCAATGAAT | (CT)_9_ | 59 | 359 | JF768529 |
| 266 | LcSSR366 | F: CGTGTGATAATACCAAAAGAGCA  R: TGTGGCACTCTCGAGTTTTT | (CT)_4_ | 59 | 217 | JF768531 |
| 267 | LcSSR367 | F: AAAGGTGTTTACCATGCTGCT  R: TCTCTCAAGACAATTGCAAGAA | (CAA)_3_…(CT)_6_ | 58 | 395 | JF768532 |
| 268 | LcSSR368 | F: TATCGGAGGCTCAATTGGTT  R: TCGAGAAATCATGAAGGAAGC | (CT)_5_…(AT)_3_ | 59 | 371 | JF768533 |
| 269 | LcSSR369 | F: CCTTAACCAAGGCTTATGCAA  R: CACAAAAACTGCAAGACACGA | (GAT)_3_…(GA)_10_ | 59 | 271 | JF768534 |
| 270 | LcSSR372 | F: CCCAATAATACAGCTTGAGTAATCT  R: GCGTGACTAACTTTTGGACA | (CTT)_4_…(GA)_7_ | 57 | 300 | JF768537 |
| 271 | LcSSR373 | F: AATCCAATTTTCACCGCTACA  R: CAAAATCGTTCTCATAGGGAAAA | (GA)_6_ta(GA)_14_ca(GA)_3_ | 59 | 399 | JF768538 |
| 272 | LcSSR376 | F: CAAAATCGTCCTCATAGGGAAA  R: TCAGGAATCCACATGTAGCAA | (CT)_30_ | 60 | 361 | JF768541 |
| 273 | LcSSR377 | F: TTGAGGGAGATTTCCAAGAAAG  R: TTTAGTCTCTCCCAAGGAAAACC | (CT)_7_…(GAA)_4_ | 59 | 383 | JF768542 |
| 274 | LcSSR379 | F: AAAATGAATTCCCGAAGAAGG  R: CAGGGCATAAAGGCACAAAT | (CT)_3_at(CT)_3_ | 59 | 388 | JF768544 |
| 275 | LcSSR381 | F: AACTCATGGCAAGGACTATTG  R: CCCTTGTATTTTCCAATCTCG | (CT)_22_…(GT)_4_gag(GT)_3_ | 58 | 240 | JF768546 |
| 276 | LcSSR382 | F: AACTCCTAGTCTCTCAAGGAACAAA  R: ATTTTCGAGACTGCCAGACA | (GA)_8_ | 59 | 383 | JF768547 |
| 277 | LcSSR383 | F: CGGCCCATATAGAAACATGA  R: ACGCAGAACCAATGCATCTA | (CT)_5_…(CTT)_4_..(CTT)_4_…(GTTT)_2_ | 59 | 395 | JF768548 |
| 278 | LcSSR384 | F: TCGATTTGATTTGGTGTGTGA  R: AATAGCATGGGAATCAGAGAGTC | (GA)_16_ | 59 | 386 | JF768549 |
| 279 | LcSSR387 | F: AAACTTTGATGAAGATCAGATTG  R: AGCGTGAGATTGGACATAAA | (CTT)_4_…(GA)_18_…(CTAT)_2_ | 55 | 367 | JF768552 |
| 280 | LcSSR388 | F: TTCGTATGAGCCGTATTTGC  R: TCAGCTCTTCCTTTCCAAACA | (ACCT)_3_…(GA)_5_…(ATTT)_2_ | 59 | 345 | JF768553 |
| 281 | LcSSR390 | F: CAAATTGAAGAAAGAAGTGACG  R: TGCCTTTTCCTAAGGACTTAAA | (CT)_9_…(GAA)_7_ | 57 | 392 | JF768555 |
| 282 | LcSSR391 | F: TTCAATCTCAGTGCTACCCACTA  R: CCGTAAAGTATGACCCATTTGA | (GT)_17_(GA)_18_ | 58 | 297 | JF768556 |
| 283 | LcSSR392 | F: TTTTCAACGGCTCCTTTGTT  R: TCACAACAGTGGTCACTCAAGAA | (CT)_13_…(GAA)_4_ | 60 | 362 | JF768557 |
| 284 | LcSSR394 | F: GAGATTCCCAAGGCAGAATA  R: CCAAGAAAGTGCTTCAAAAAG | (CT)_13_…(GAA)_3_ | 56 | 265 | JF768559 |
| 285 | LcSSR395 | F: TTTTCAACGGCTCCTTTGTT  R: TGCTTCAAAAAGCTGTATCACAC | (AT)_4_…(CT)_9_…(GAA)_3_ | 59 | 300 | JF768560 |
| 286 | LcSSR397 | F; TCTCAAGGAACAAACCAGCA  R: TGGGATCAAGTGATACTTTTGG | (AAAT)_2_…(CTTT)_3_…(GA)_9_ | 59 | 345 | JF768562 |
| 287 | LcSSR399 | F: CAATGGAACCACTTAAACAACC  R: GCCTTTTCAACGGCTACTTT | (CTT)_4_…(GA)_10_ | 58 | 469 | JF768564 |
| 288 | LcSSR402 | F: TTCTGGGAACAGCAGAAAAGA  R: AAATGCACGTCAAAACCTGA | (CT)_10_ | 59 | 267 | JF768567 |
| 289 | LcSSR403 | F: GCCTTTTCAACGGCTAAAAT  R: TGCTTGAGAAAACTGACACACA | (CT)_10_…(GAA)_5_…(ATT)_3_ | 59 | 422 | JF768568 |
| 290 | LcSSR404 | F: CCGTTTAATTCTTCTTGAGAATCC  R: GGAACAAGTGGTATATTTGGAGAA | (CTT)_5_…(GA)_14_ | 59 | 390 | JF768569 |
| 291 | LcSSR406 | F: TTTTCAACGGCTCCTTTGTT  R: AACCGTGGTCTCTCAAGACTATT | (CA)_4_…(CT)_7_…(ATT)_3_ | 59 | 377 | JF768571 |
| 292 | LcSSR408 | F: TTTTCAACGGCTCCTTTGTT  R: AACCGTGGTCACTCAAGACTATT | (CCAA)_2_…(CT)_7_…(GAA)_3_ | 59 | 366 | JF768573 |
| 293 | LcSSR409 | F: GCCTTTTTCAACGGCTACTTT  R: AACCGTGGTCACTCAAGACTATT | (CT)_8_…(GAAA)_2_…(ATTT)_2_ | 59 | 380 | JF768574 |
| 294 | LcSSR410 | F: CAGCGCAAGATTGGACATAA  R: TGCTTACGCGTGAACTAACAA | (CT)_7_ | 59 | 391 | JF768575 |
| 295 | LcSSR412 | F: TTTTCAACGGCTCCTTTGTT  R: AACCGTGGTCTCTCAAGACTATT | (CT)_10_…(GAA)_3_ | 59 | 362 | JF768577 |
| 296 | LcSSR413 | F: TGGAACAACCACTTAGACAACTG  R: CAACACTCATCACACTTACGAGAA | (GA)_10_ | 59 | 346 | JF768578 |
| 297 | LcSSR414 | F: TTGCTTCAACAAGCTATATCACAAC  R: GCCTTTTCAACGGCTACTTT | (CTT)_5_…(GA)_8_ | 59 | 275 | JF768579 |
| 298 | LcSSR415 | F: TCAAAAAGCTGTATCACAACAGTG  R: TGGGAGATTTCCAAGACAGA | (CTT)_4_…(GA)_11_ | 59 | 256 | JF768580 |
| 299 | LcSSR416 | F: CCTTTTCAATGGCTACTTTGG  R: TGAGAAATCGGACACACAAC | (CT)_18_…(GAA)_4_ | 58 | 400 | JF768581 |
| 300 | LcSSR417 | F: CAAGACTCTTCTAGCCTTTTCAA  R: TTTGCTTCAACAAGCTGTACC | (CT)_10_…(GAA)_5_ | 58 | 288 | JF768582 |
| 301 | LcSSR418 | F: TCTTGCTTACGCGTGGACTA  R: CAAATTGAAGAAAGAAGCAACG | (CTT)_4_…(GA)_8_ | 59 | 197 | JF768583 |
| 302 | LcSSR420 | F: CATTTAGAAATGAAGGGAGCTAA  R: TCTTTCACATCGGAAAAAGTT | (GA)_14_ | 56 | 298 | JF768585 |
| 303 | LcSSR422 | F: CAAAATCGTTCTCTTACGGATAA  R: GAATTTTGTGTGTTACATGATTAGC | (CT)_13_ | 57 | 428 | JF768587 |
| 304 | LcSSR424 | F: GCGGAAGGAATGGTAAATAA  R: TCATGTTCTTCACTTGTATTCTCTC | (CTAT)_7_ccat(CTAT)_8_…(CT)_5_…(CT)_23_ | 56 | 293 | JF768589 |
| 305 | LcSSR426 | F: TCCAACTGATTACAATGGAACAAC  R: CAACGCTCACATTAAGAAAGCA | (GT)_8_(GA)_14_ | 60 | 371 | JF768591 |
| 306 | LcSSR428 | F: GCTTGAGTAATCTGACTCACAACTG  R: TGGGAGTTTTCCAAGACTGAA | (CTT)_3_…(GA)_10_ | 59 | 343 | JF768593 |
| 307 | LcSSR429 | F: ACCCCTAGTCTCTCAAGGAACA  R: TTGGGAAAACAACTTTTGCTG | (GA)_20_aa(GA)_16_ | 60 | 377 | JF768594 |
| 308 | LcSSR430 | F: TCAGCACACGATTGGACATAA  R: CAAGAGATGCTTCAAAAAGCTG | (CT)_11_…(GAA)_3_ | 60 | 382 | JF768595 |
| 309 | LcSSR431 | F: AGGGCAAAAACACAAACGAC  R: AAGGCTTCGCCAGAAAAAGT | (GA)_9_ | 60 | 371 | JF768596 |
| 310 | LcSSR432 | F: CAAGACTAATCTAGCCTTTTCAACG  R: AACCGTGGTCACTCAAGACTATT | (CT)_11_ | 59 | 400 | JF768597 |
| 311 | LcSSR433 | F: CCTTCATTTTTCAACCTGCAA  R: TTCGAACACTCTCTGTTTGTTGA | (CT)_9_ | 60 | 400 | JF768598 |
| 312 | LcSSR434 | F: AAGAACAAATATGGGCAAAAGG  R: CTTGCTTACGCGTGGGACTA | (CT)_5_..(CT)_5_ | 61 | 235 | JF768599 |
| 313 | LcSSR435 | F: TGGGAGATTTCCAAGACAGAA  R: TTTCTTGAATATGAACGTGAGAGAG | (CT)_20_ | 59 | 207 | JF768600 |
| 314 | LcSSR436 | F: GGGAGTCTTTCAAGGATCAA  R: GTTGATAAGTTAAGATGCAGAAGC | (CT)_7_ | 56 | 250 | JF768601 |
| 315 | LcSSR437 | F: GAGTTGCTTCAAAAAGCTATATCAC  R: TTTTCAACGGCTCCTTTGTT | (CTT)_4_…(GA)_9_ | 59 | 286 | JF768602 |
| 316 | LcSSR438 | F: TCGAGTCATGGTTTGCACTT  R: GAAACGAAATCAATCACACACC | (CT)_10_ | 59 | 383 | JF768603 |
| 317 | LcSSR439 | F: GCCTTTTCAACGGCTAATTTC  R: TTGATGAAGATCAGAGTGTTCTTGT | (CT)_23_…(GAA)_3_ | 60 | 320 | JF768604 |
| 318 | LcSSR440 | F: CAAAGATTGCTTCAAAAAGCTG  R: TGCTAGCCTTTTCAAAACCAG | (CTT)_3_…(GA)_11_ | 59 | 297 | JF768605 |
| 319 | LcSSR441 | F: AAAGACGTTTACCATGCTGCT  R; AACCGTGGTCTCTCAAGACTATT | (GAA)_3_…(CT)_7_…(GAA)_3_ | 58 | 400 | JF768606 |
| 320 | LcSSR442 | F: GCCTTTTCAACGGCTACTTT  R: TGCAATAAACCTGCTTGAGAAA | (CT)_8_ | 59 | 392 | JF768607 |
| 321 | LcSSR443 | F: TGATAAGTTGATAAGATGCAGAAGC  R: CAAAACACCCAACGACTTTTC | (GT)_8_(GA)_11_ | 59 | 347 | JF768608 |
| 322 | LcSSR444 | F: AAAAGATCAATGTGCGTGTCC  R: AACGTGCCAATAACACTTTCAA | (CA)_4_ | 59 | 379 | JF768609 |
| 323 | LcSSR445 | F: TTCGTTCCCTTTCCAAAATG  R: TTGCAAGGAAATGTGCAGAG | (GTGA)_3_(GA)_14_ | 59 | 393 | JF768610 |
| 324 | LcSSR446 | F: GTGTGTGTACAAAGATTTGCTTCT  R: TTGTGGGAGTTTTTCCAAGA | (CTT)_3_…(GA)_9_ | 58 | 289 | JF768611 |
| 325 | LcSSR447 | F: CAACATCATTCTCATAGGGAAAA  R: ATTGATCCAACATCGAAGCA | (CT)_11_ | 59 | 246 | JF768612 |
| 326 | LcSSR448 | F: TTTTGGGAGTTTTCCAAGGT  R: GCAAAGGTTGCTTCAAAAAG | (CT)_5_…(ATT)_4_ | 58 | 275 | JF768613 |
| 327 | LcSSR449 | F: ATTGTGGGAGTTTTCCAAGG  R: GAGTTGCTTCAAAAAGCTATATCAC | (CT)_11_(CA)_4_…(GAA)_3_ | 58 | 278 | JF768614 |
| 328 | LcSSR450 | F: GCCCTAGTCTCTCAAGGAACAA  R: TGGGAGTTTTCCAAGACTGAA | (CTT)_3_…(GA)_13_ | 59 | 374 | JF768615 |
| 329 | LcSSR451 | F: CAAAGGTGTGTACAGAAATGTGC  R: CGTCAATTCACTCCATGCTG | (CTT)_4_…(GA)_9_ | 60 | 360 | JF768616 |
| 330 | LcSSR452 | F: CGCTATGCTTCCCAAGAATC  R: CAGCTGGGACTTTCAATGTG | (GA)_13_ | 59 | 399 | JF768617 |
| 331 | LcSSR453 | F: GCTGTATCACTAACTGTGATGTTTT  R: GCTCCTTTGTGGAAGAAAGA | (CTT)_3_…(GA)_14_ | 57 | 289 | JF768618 |
| 332 | LcSSR454 | F: CAAAGACTTTCAACACTTTGTCG  R: AAAGGTGTTTACCATGCTGCT | (CTT)_4_…(GA)_11_ | 58 | 399 | JF768619 |
| 333 | LcSSR455 | F: CAGATTATTATATTGGAACAAGTGG  R: ATCACAACAGTGATGTTTTACACT | (CT)_18_…(GAA)_3_ | 56 | 249 | JF768620 |
| 334 | LcSSR456 | F: GCTTCTAAAAGCTATATCACAACAG  R: TGGACATAAAGGTGTTTACCAT | (AAT)_3_…(CTT)_3_…(GA)_9_aa(GA)_13_ | 56 | 400 | JF768621 |
| 335 | LcSSR457 | F: TCAACGGCTCCTTTGTTAGAA  R: TGCTTCAAAAAGCTATATCACAAAC | (GAA)_4_…(CT)_9_…(GAA)_3_ | 59 | 300 | JF768622 |
| 336 | LcSSR458 | F: TGCAATATGACATGGCTCTCA  R: CAGGAAATACTCGTCGGAAAA | (CCT)_3_…(CT)_6_ | 60 | 297 | JF768623 |
| 337 | LcSSR459 | F: TCCTGACTAAAGCCCTAGTATCTCA  R: TTGTGGGGAGATTTCCAAGA | (CTT)_4_…(GA)_9_ | 60 | 388 | JF768624 |
| 338 | LcSSR460 | F: CAACTCTAGTCTCTCAAGGAAAACT  R: GCTAACGCTTTTATAACAAATCTC | (GA)_9_ | 56 | 300 | JF768625 |
| 339 | LcSSR461 | F: TGGTTCGTTGACCAAACTGT  R: ATTGTGGGAGATTTCCAAGG | (CTT)_4_…(GA)_9_ | 59 | 498 | JF768626 |
| 340 | LcSSR462 | F: GAGTTGCTTCAAAAAGCTATATCAC  R: AAAGACGTTTACCATGCTGCT | (GA)_7_ | 58 | 349 | JF768627 |
| 341 | LcSSR463 | F: GCTGTAATCACAACCGTGATATT  R: CAAACCACAACTTTTACACAACG | (GT)_5_(GA)_5_gg(GA)_6_ | 59 | 244 | JF768628 |
| 342 | LcSSR464 | F: TGGACAAGAGTACATGCAGA  R: CAAAGATTGCTTCAAAAAGTTG | (CT)_8_…(GAA)_4_ | 56 | 399 | JF768629 |
| 343 | LcSSR465 | F: GCCTTTTCAACGGCTACTTT  R: AACCGTGGTCTCTCAAGACTATT | (CT)_7_cc(CT)_6_…(GAA)_5_ | 58 | 384 | JF768630 |
| 344 | LcSSR466 | F: ATTTCCGGGAGATTTCCATC  R: AAACTCCAACCCTTCACACAA | (GA)_4_..(GA)_8_ | 60 | 336 | JF768631 |
| 345 | LcSSR467 | F: CATACTCAGGAGACTTACGGTGTTA  R: CATGAAGAGCCACCATGAAA | (GA)_4_ca(GA)_18_ta(GA)_6_ | 59 | 399 | JF768632 |
| 346 | LcSSR468 | F: CAGCGCAAGATTGGACATAA  R: CAAAGATTTGCTTCTAAAAAGCTG | (CT)_7_…(GAA)_3_ | 59 | 371 | JF768633 |
| 347 | LcSSR469 | F: TCTCAAGGAACAAACCAGCA  R: TGGGAGTTTTCCAAGACTGAA | (CTT)_4_…(GA)_8_…(AT)_4_ | 59 | 371 | JF768634 |
| 348 | LcSSR470 | F: GTGTTACAGAAAAGCTGAAAAGTTC  R: TTGAGGGAGATTTCCAAGAC | (GA)_9_ | 57 | 341 | JF768635 |
| 349 | LcSSR471 | F: GAGTTGATTCAAAAAGCTATATCAC  R: CAGCGCAAGATTGGACATAA | (CTT)_4_…(GA)_14_ | 59 | 382 | JF768636 |
| 350 | LcSSR472 | F: TTGATGATGATCAGATTGTTCTTG  R: AAAGGTGTTTACCATGCTGCT | (CTT)_4_…(GA)_10_ | 59 | 326 | JF768637 |
| 351 | LcSSR473 | F: CTTTGATGATGATCAGTTGTTCTTG  R: AACAAGTTTTCTGCACGTGTGT | (CTT)_4_…(GA)_17_ | 60 | 575 | JF768638 |
| 352 | LcSSR474 | F: AAACTTGATGAAGACCAGAGTG  R: ATCTCCTAGCCTTTTCAACG | (GA)_11_ | 56 | 300 | JF768639 |
| 353 | LcSSR475 | F: CAAGACAATTGCAAGAAACC  R: GAACAAGTGGTATATTTGGAGAAG | (AAT)_3_…(CTT)_4_…(GA)_16_ | 56 | 300 | JF768640 |
| 354 | LcSSR476 | F: CCTTTTCAACGGCTAATTTCC  R: TTGATGAAGATCAGAGTGTTCTTGT | (CT)_9_…(GAA)_3_ | 59 | 284 | JF768641 |
| 355 | LcSSR477 | F: GCTGTATCACTAACTGTGATGTTTT  R: CTGGATCAAGTGATACTTTTGGA | (CTT)_3_…(GA)_11_ | 58 | 231 | JF768642 |
| 356 | LcSSR478 | F: CTGTGGGATATTTCCAAGACAA  R: TGTTCTTGTAAAACTTTGATGATGC | (CAA)_3_…(CT)_7_…(GAA)_3_ | 59 | 229 | JF768643 |
| 357 | LcSSR479 | F: TTTCAACGGCTCCTTTATGG  R: GCGGGATAACACTCTTGCTT | (CT)_7_ | 60 | 328 | JF768644 |
| 358 | LcSSR480 | F: TGCTAGCCAATACAAAACACTCA  R: GATGCAAAAGCTTGTTCTTCAG | (CT)_8_ | 59 | 315 | JF768645 |
| 359 | LcSSR481 | F: GAATGTGCTTCAAAAAGCTTCA  R: CTTTTCAACGGCTGCTTTGT | (CTT)_4_…(GA)_9_ | 60 | 289 | JF768646 |
| 360 | LcSSR482 | F: AGCCTTTTCAACGACTACTTTG  R: TTGCTTCAAAAAGCTGTATCAC | (CT)_10_…(GAA)_4_ | 57 | 295 | JF768647 |
| 361 | LcSSR483 | F: CGGCTACTTTGTGAGAAGAAAGA  R: TGCTTGGGAAATCTGACACA | (CT)_15_…(GAA)_5_ | 60 | 396 | JF768648 |
| 362 | LcSSR484 | F: AGTCTCTCAAGGAACAATGCAA  R: TCAACGGCTCCTTTGTTAGAA | (CTT)_5_…(GA)_9_ | 59 | 400 | JF768649 |
| 363 | LcSSR485 | F: ACCACACGCATGACATTGTT  R: TGAGCTAACTTCATTCCTGGTT | (AT)_6_ | 59 | 279 | JF768650 |
| 364 | LcSSR486 | F: AGACTACGTTAGCCTTTTCAACG  R: TCACAAAGTGATGTATTACACAGAA | (CTAT)_2_(CT)_6_ | 59 | 273 | JF768651 |
| 365 | LcSSR487 | F: CAATGGAACCACTTAAACAACC  R: TTTCAACGGCTCCTTTTGTT | (CTT)_3_…(GA)_11_ | 59 | 483 | JF768652 |
| 366 | LcSSR488 | F: AAAGGTGTTTACCATGCTGCT  R: CTCTCACGGAACAAATGCAA | (CT)_13_…(GAA)_3_ | 59 | 476 | JF768653 |
| 367 | LcSSR489 | F: AGAGATGCGCTGGAAAATCT  R: CATCACCTGCAGAAAGCAAA | (GA)_8_..(GA)_4_ | 59 | 466 | JF768654 |
| 368 | LcSSR490 | F: CAACCGTTTAAGCCTTCTTGA  R: TTTTCAACGGCTCCTTTGTT | (AAT)_3_…(GA)_7_ | 59 | 442 | JF768655 |
| 369 | LcSSR491 | F: GAAACTCTAGTCTGTCAAGGAAAA  R: CAACACTCATAACACTTACGAGAAA | (GA)_8_…(GTTT)_2_ | 57 | 294 | JF768656 |
| 370 | LcSSR492 | F: TCAAACAACAACAACTACAACACTC  R: GCAACTCTAGTCTCTCAAGGAAAA | (CT)_5_ | 58 | 299 | JF768657 |
| 371 | LcSSR493 | F: TGAGAATCCTGACTAAAGCCCTA  R: TGGGAGATTTGCAAGACTGA | (CTT)_3_…(GA)_8_ | 59 | 394 | JF768658 |
| 372 | LcSSR494 | F: TGTCAGCGTAAGATTGGACA  R: GCTTCTAAAAGCTATATCACAACAG | (CT)_16_…(GAA)_3_ | 57 | 375 | JF768659 |
| 373 | LcSSR495 | F: TTTTCAAACGGATACTTTCCA  R: CTTCAAATGATAAGTTGATAGATGC | (CT)_8_ | 57 | 293 | JF768660 |
| 374 | LcSSR496 | F: TATAGGGCGCTCACATGTAT  R: CAATAACCACGGGAAAAAT | (CA)_4_ | 55 | 400 | JF768661 |
| 375 | LcSSR497 | F: GCACGAGATGGGACAAAAAT  R: TCTTTCTTGAATATGAACGTGAGAG | (CT)_11_ | 59 | 296 | JF768662 |
| 376 | LcSSR498 | F: TTGATGAAGATCAGAGTGTTCTTGT  R: GCCTTTTCAACGGCTAATTTC | (CTT)_4_…(GA)_14_…(GTT)_3_ | 60 | 296 | JF768663 |
| 377 | LcSSR499 | F: GCCTTTTCAACGGCTACTTT  R: TGCTTGAGAAATCTGACACACA | (CT)_6_..(CT)_7_…(GAA)_8_ | 59 | 393 | JF768664 |
| 378 | LcSSR500 | F: TGTGTCAGCACAAGATTGGA  R: CGAATGCAATAAACCTGCTG | (CT)_7_ | 59 | 458 | JF768665 |
| 379 | LcSSR501 | F: TGCTTGAGAAATCTGACACACA  R: TGGACAAGAGTACGTTTACACCA | (CTT)_4_…(GA)_9_…(GT)_4_…(GTT)_3_ | 59 | 454 | JF768666 |
| 380 | LcSSR502 | F: TCAAGGAAACCCTTATCTTAAACC  R: CGGTGTCTTATCATGCTGCT | (GAT)_3_…(CTT)_4_…(GA)_13_ | 59 | 485 | JF768667 |
| 381 | LcSSR503 | F: CAAACTGACTTACAATGGAACCAC  R: GCCTTTTCAACGACTCCTTT | (GA)_8_ | 59 | 480 | JF768668 |
| 382 | LcSSR504 | F: TGCTTCTAAAAGCTGTATCACAACA  R: GCCTTTTCAACGGCTAATTTC | (AAT)_3_…(CTT)_3_…(GA)_20_ | 60 | 328 | JF768669 |
| 383 | LcSSR505 | F: TCAAACACAACACTCACGAGAA  R: TGGAGCAACCGTTTAAACAAC | (CT)_8_ | 60 | 349 | JF768670 |
| 384 | LcSSR506 | F: CGGTTACTTTCCATTGGATGA  R: TGATAAGAGTTGATGAAGTGCAGA | (CT)_13_ | 59 | 318 | JF768671 |
| 385 | LcSSR507 | F: TCAGCACACGATTGGACATAA  R: GGTCACTCAAGACTATTGCAAACA | (CT)_7_ | 60 | 398 | JF768672 |
| 386 | LcSSR508 | F: ACAAAATCGTTCTCATACGG  R: CATTTAGAAATGAAGGGAGCTAA | (CT)_14_ | 56 | 304 | JF768673 |
| 387 | LcSSR509 | F: GGATTGGACGTGAAGACGTT  R: CAAGAGTTGCTTCAAAAAGCTG | (CT)_10_…(GAA)_3_ | 59 | 364 | JF768674 |
| 388 | LcSSR510 | F: CAAGAATGTGTACAAAGGTTGC  R: AAAGACGTTTACCATGCTGCT | (CTT)_4_…(GA)_10_ | 58 | 325 | JF768675 |
| 389 | LcSSR511 | F: TGATAAGTTGATAAGATGAAGAAGC  R: TTGGGAATATTTCAAGGATCTACA | (GA)_10_..(GA)_4_ | 58 | 273 | JF768676 |
| 390 | LcSSR512 | F: AAAAAGCTTTATCACAAAGTGATGT  R: AAAGTTAATTTCTCCATGCTGCT | (CTT)_3_…(GA)_8_ | 58 | 348 | JF768677 |
| 391 | LcSSR513 | F: AAAGCCAAAGCCACACAAAT  R: TTGGGAAGTGGAATGGTGTT | (CT)_4_..(CT)_4_…(CAT)_3_ | 60 | 325 | JF768678 |
| 392 | LcSSR514 | F: CTCTCAAGGAACAAATGCAA  R: TGGGAGATTTTCAAGACAGATT | (CTT)_4_…(GA)_10_ | 57 | 380 | JF768679 |
| 393 | LcSSR515 | F: AACCGCATGCATAAGAGTCA  R: GAAAAATGTTGTGTAAGGGGTGA | (GT)_4_ | 60 | 229 | JF768680 |
| 394 | LcSSR516 | F: CAAGACTACGCTAGCCTTTTCAA  R: AACCGTGGTCACTCAAGACTATT | (CT)_11_…(GAAA)_2_ | 60 | 397 | JF768681 |
| 395 | LcSSR517 | F: TGATTAAACTCGGATACACGAT  R: ATAGCCCAAACCCATGATTA | (GA)_4_..(GA)6(GT)_7_ | 56 | 300 | JF768682 |
| 396 | LcSSR518 | F: TCAGCACACGATTGGACATAA  R: CAAGTGTTGCTTCAAAAAGCTG | (CT)_7_…(GAA)_5_ | 60 | 365 | JF768683 |
| 397 | LcSSR519 | F: TACGCTAGCCTTTTCAACGA  R: AACCGTGGTCACTCAAGACTATT | (CT)_10_…(GAAA)_2_ | 59 | 392 | JF768684 |
| 398 | LcSSR520 | F: TGCAATAAACCTGCTTGAGAAA  R: CAACGCTACTTGGTAGGAAGAAA | (AAT)_3_…(GA)_9_ | 59 | 396 | JF768685 |
| 399 | LcSSR521 | F: TTGCAGTCTTCATCAGAAGGA  R: TGTCTTGGAAAACTCCCACA | (GAA)_3_ | 59 | 244 | JF768686 |
| 400 | LcSSR522 | F: TGTGGGAGTTTTCCAAGACA  R: CAAATGCAATAAACCTGCTTGA | (CT)_7_…(GAA)_4_ | 60 | 358 | JF768687 |
| 401 | LcSSR523 | F: CAACTCTTACACAACATTTGCTGA  R: AGCAACTCTAGTCTCTCAAGGAACA | (CT)_11_ | 60 | 284 | JF768688 |
| 402 | LcSSR524 | F: CACCATGTAGTGTGTCAACATGA  R: CGCAAGCTTAAATCCAATGC | (CT)_8_ | 60 | 420 | JF768689 |
| 403 | LcSSR525 | F: TTGAGAATCCTGACCAACACC  R: AGTGTCTTCGTGGCTTGTGA | (GA)_11_ | 59 | 385 | JF768690 |
| 404 | LcSSR526 | F: TCTCAAGGAACACCAATCTTAAAC  R: CATTTGGCATCAAGAAACAATC | (GA)_7_ | 59 | 250 | JF768691 |
| 405 | LcSSR527 | F: TGTTTCTTGATGCCAAATGTTC  R: TGTGTCAGCATGAGATTGGA | (AT)_5_ | 59 | 289 | JF768692 |
| 406 | LcSSR528 | F: TCTCGCCAATACAACTCGAA  R: TCCCACCTGCAGTAGAAAAA | (GA)_9_ | 59 | 381 | JF768693 |
| 407 | LcSSR529 | F: GCCTTTTCAACGGCTACTTT  R: TCACAGAAGTGATGTTTTACACAAA | (CT)_8_…(GAA)_3_ | 58 | 282 | JF768694 |
| 408 | LcSSR530 | F: TGATGAAGATCAGAGTTGTTCTTGT  R: TTATGCCATGCAGTGTGTCA | (CTT)_3_…(GA)_9_…(GTT)_3_ | 59 | 350 | JF768695 |
| 409 | LcSSR531 | F: TCCATGACTTTTCAACGGTTA  R: TCAGAACTTGTTCTTGAGTTTCTTG | (AT)_5_…(ATTT)_2_…(CT)_8_ | 59 | 274 | JF768696 |
| 410 | LcSSR532 | F: CTTTGAACATCTATCAAGCGTACAA  R: GCCCTAGTCTCTCAAGGAACAA | (CT)_7_…(GAA)_3_ | 59 | 282 | JF768697 |
| 411 | LcSSR533 | F: GATCAAATGGGCAAGAGGAA  R: GGTTCCATTGTAAATCAGGTTGA | (CACAA)_3_ | 60 | 248 | JF768698 |
| 412 | LcSSR534 | F: ACGACTGACGTAACAGGAATTGT  R: TTAATCCCATGGAGAGGAACA | (GAAA)_3_ | 59 | 238 | JF768699 |
| 413 | LcSSR535 | F: GCCTCTTCTCCAGCTTCTT  R: CGTCAATGAAAAGTCAGATCA | (CT)_4_…(CT)_3_ | 56 | 250 | JF768700 |
| 414 | LcSSR536 | F: CGGTTAAGTGTTATGCACCA  R: TTTGGTTTAGTGGATTGAAGC | (CACAA)_3_ | 57 | 233 | JF768701 |

**S1B Table. List of *L. culinaris* SSR primer pairs developed from the (GAA)_14_ enriched microsatellite library. The primer sequences (F/R), microsatellite repeat motif structure, annealing temperature (Tm), expected size of the amplification product (bp), along with the GenBank accession numbers are mentioned.**

| **S.No.** | **Locus** | **Sequence F: Forward (5’-3’), R: Reverse (5’-3’)** | **Repeat motif** | **Tm (ºC)** | **Product Size (bp)** | **Accession No.** |
| --- | --- | --- | --- | --- | --- | --- |
| 1 | LcSSR601 | F:TGCTTCACCTGCTTGAAAGA  R:TTGGATGATGGAAGCTAGTGG | (CTT)_5_…(CTT)_3_ | 60 | 378 | KJ470796 |
| 2 | LcSSR602 | F:AGAAAAGCCATCAGTCCACAA  R:GCACACATCACAGCAAATCC | (GAA)_7_...(GAA)_3_…(GAA)_3_ | 60 | 374 | KJ470797 |
| 3 | LcSSR603 | F:GCAGATGAAGATGTTGCCATT  R:ACGCTCATACGCTCATACTCCT | (GAA)_8_...(GAA)_3_...(GAA)_3_...(GAA)_4_...GAA)_6_ | 60 | 469 | KJ470798 |
| 4 | LcSSR604 | F:TCACGAACATTCAGGTTTTGAC  R:TATGGAACTTGTCCCCCACT | (GAA)_10_ | 60 | 399 | KJ470799 |
| 5 | LcSSR605 | F:CCTGAGTAAGATGTGCAGCAA  R:CATGTGGCCCTAAGTCCATT | (GAA)_9_ | 59 | 380 | KJ470800 |
| 6 | LcSSR606 | F:GAACTCAATGAGACCGATCTTT  R:AACCCAAATTTCCCCCTAAA | (GAA)_6_ | 59 | 400 | KJ470801 |
| 7 | LcSSR607 | F:CACAACCATCTCAACAATCTCA  R:TTTTAACTCGCCCTGTGACC | (GAA)_12_ | 58 | 312 | KJ470802 |
| 8 | LcSSR608 | F:GCCTTGGGACTCTTCTTCAA  R:TTTCTGTTATTACCCTTGGTGT | (GAA)_7_ | 59 | 388 | KJ470803 |
| 9 | LcSSR609 | F:ACCATGCAGTGTGTCAGCAT  R:CTTGCTTTACGCGTGGACTA | (CT)_7_..(GAA)_6_...(ATT)_3_ | 60 | 396 | KJ470804 |
| 10 | LcSSR610 | F:AAAGCTTTATCACAACAGTG  R:GACTACTCTAGCCTTTTCAA | (CTT)_6_...(GA)_9_ | 50 | 298 | KJ470805 |
| 11 | LcSSR611 | F:TTTATGGCATGGAGTGTGTCA  R:TTCCTAAGATGTTTAACGTTGG | (CT)_29_ | 59 | 444 | KJ470806 |
| 12 | LcSSR612 | F:TCCCTTGAGAGATGAAGTTTCC  R:CGAAAAGTTGCATTGCTTTG | (GAA)_9_ | 59 | 281 | KJ470807 |
| 13 | LcSSR613 | F:ACCGTTGGCTGGTTTAGATG  R:TTCCAACTGCTTCTTTAATTCG | (GAA)_6_ | 59 | 397 | KJ470808 |
| 14 | LcSSR614 | F:TTTTCCTTCCTTATTTGGTTCC  R:TTCCACTTGGAAACACATTCC | (GAA)_9_...(GAA)_4_ | 59 | 367 | KJ470809 |
| 15 | LcSSR615 | F:TGGATCAAGTGGTATATTTGGA  R:TCTCTCAAGGAACAAATGCAC | (CT)_8_...(GAA)_6_ | 60 | 297 | KJ470810 |
| 16 | LcSSR616 | F:AAGTTTTGTTCCCTTCTAGT  R:AATGATCTTCATCTTCTTCC | (CT)_11_...(CTT)_8_ | 50 | 353 | KJ470811 |
| 17 | LcSSR617 | F:CTCAAGACAATTGCAAGAAACC  R:TGTGGGATATTTCCAAGACAGA | (CTT)_8_...(GA)_11_ | 59 | 324 | KJ470812 |
| 18 | LcSSR618 | F:GGGTTACGACTCAAGCTCAAA  R:TGGCGTCTCTCCATTTTATG | (GAA)_11_ | 59 | 400 | KJ470813 |
| 19 | LcSSR619 | F:ACCAGACACATTGGGCATTT  R:GGGCCCTGGAAAATATGACT | (GAA)_11_ | 60 | 356 | KJ470814 |
| 20 | LcSSR620 | F:AAAGCTTTATCACAACAGTG  R:CTATTCTAGCCTTTTCAACA | (CTT)_6_...(GA)_15_ | 50 | 326 | KJ470815 |
| 21 | LcSSR621 | F:CTTTGAGAGACCACAATTT  R:GACGTTTAAGCATAACAAAC | (GAA)_4_...(GAA)_6_ | 50 | 357 | KJ470816 |
| 22 | LcSSR622 | F:CCGCGTTCATAAAGGAAGTT  R:CCATTGATCCTTGAATCTTTC | (GAA)_9_ | 59 | 379 | KJ470817 |
| 23 | LcSSR623 | F:TCCATCTTCTACTGGCATTG  R:GGTTGTGTTGCGTCTTCTACTG | (GAA)_7_ | 59 | 400 | KJ470818 |
| 24 | LcSSR624 | F:CCCCAAAGGACTCATACACA  R:CCTTATCTGGAGAGCATTTTTG | (AT)_10_ | 58 | 398 | KJ470819 |
| 25 | LcSSR625 | F:CTTGGTGGTGGAAGGATGTT  R:ATAACAAGAGGCCACCGAGA | (CTT)_6_ | 59 | 423 | KJ470820 |
| 26 | LcSSR626 | F:TCCTACCGTAAAAGGGATCAGA  R:GCGTGGACTAACCAAGGAAA | (GAA)_7_ | 60 | 299 | KJ470821 |
| 27 | LcSSR627 | F:TCAAATTCATTGCTCTTCCTCT  R:GCGTTCATGCTCAGAAACAA | (CTT)4...(CTT)5(GTT)3 | 60 | 379 | KJ470822 |
| 28 | LcSSR628 | F:TCCATTCGTAATCATGTCTTTG  R:GATGTTGGGGGCTAAACCTT | (CT)_21_...(CTT)_10_ | 60 | 399 | KJ470823 |
| 29 | LcSSR629 | F:TCCTCTTCCAACCTCAGGTAA  R:CCTGATAAATGCCATCACCA | (GAA)_9_ | 59 | 246 | KJ470824 |
| 30 | LcSSR630 | F:CTCTCAAGGAACAAATGCAA  R:TGGGAGATTTCCAAGACAAGA | (CTT)_7_... (GA)_7_ | 59 | 364 | KJ470825 |
| 31 | LcSSR631 | F:GCGACAAAGGCTTAAAACCA  R:AAACATGAGGGAAAATGTGGA | (CT)_8_...(CA)_7_ | 60 | 393 | KJ470826 |
| 32 | LcSSR632 | F:TGTCATCTTCTTAGGGAATA  R:ATGATGAAAGATGACCAGT | (GAA)_3_...(GAA)_5_...(GAA)_3_ | 50 | 295 | KJ470827 |
| 33 | LcSSR633 | F:AATTGTGGACAACGTGCAGA  R:TTGTGACCATCAACAATGCTT | (GAA)_7_ | 60 | 423 | KJ470828 |
| 34 | LcSSR634 | F:TGCTCTCTTCTCCTTCAACCT  R:AAATGATGGTTGCAAATGGA | (CAA)_3_...(GAA)_6_ | 58 | 379 | KJ470829 |
| 35 | LcSSR635 | F:AGTCTCTCAAGGAACAAATTGC  R:TGTGGGAGATTTCCAAGACAG | (CTT)_7_...(GA)_6_ | 60 | 367 | KJ470830 |
| 36 | LcSSR636 | F:GGGTTTTAAAGTTGGGTTGGA  R:CAAAATTCGCGTATGTTAATCG | (GAA)_6_ | 60 | 383 | KJ470831 |
| 37 | LcSSR637 | F:TGGGAGATTTCCAAGACAGAA  R:AACCGTGGTCTCTCAAGACAAT | (CT)_4_...(GAA)_7_ | 60 | 300 | KJ470832 |
| 38 | LcSSR638 | F:GCTGGGTTGGATGTTGTTTT  R:TCTACTTCTCTCTCTGGCGGTA | (GAA)_6_ | 59 | 293 | KJ470833 |
| 39 | LcSSR639 | F:GCCCCTCAAGGAACTTAATCA  R:TTTTGTCTCATTCTCTCAACCA | (CTT)_6_ | 60 | 400 | KJ470834 |
| 40 | LcSSR640 | F:TGTTTGCGTTGAGATCATGG  R:CAATGATCGGACCTTGGAGT | (CTT)_6_ | 60 | 388 | KJ470835 |
| 41 | LcSSR641 | F:AAACTCATTGTTTCTGTTGTGC  R:TGCAGTTGTCGGAATATGCT | (CAA)_3_... (GAA)_7_ | 59 | 352 | KJ470836 |
| 42 | LcSSR642 | F:GTTTTATCTTTACGCCATC  R:AAAGCTTTATCACAACAGTG | (CT)_11_... (GAA)_6_ | 50 | 438 | KJ470837 |
| 43 | LcSSR643 | F:TGTGGGAGATTTCCAAGACTG  R:CTCAAGGAACAAACCGCAAT | (CT)_9_...(GAA)_8_ | 60 | 378 | KJ470838 |
| 44 | LcSSR644 | F:ATATTAGCAAGTGGACAAGA  R:AAAGCTTTATCACAACAGTG | (CT)_7_...(GAA)_7_ | 50 | 399 | KJ470839 |
| 45 | LcSSR645 | F:TTGGTTGATCATGTGGATGAG  R:TCGATATCACGAACCCAATTC | (GTT)_4_...(GTT)_8_ | 59 | 390 | KJ470840 |
| 46 | LcSSR646 | F:GATCTATGCTCAGCTCCCAGAT  R:TCTCCTTAGTCACTGTTCATCA | (GAA)_7_... (GAA)_8_ | 59 | 397 | KJ470841 |
| 47 | LcSSR647 | F:CCTGAGGGGAACAAAGTGTTT  R:CCGTTCAGCAGTTTTCTCCT | (CTT)_7_ | 60 | 393 | KJ470842 |
| 48 | LcSSR648 | F:ACACAACCGTGGTCTCTCAA  R:TGGATCAAGTGGTATATTTGGA | (AAT)_3_...(CTT)_6_...(GA)_7_ | 59 | 292 | KJ470843 |
| 49 | LcSSR649 | F:AAAAGGAGAGCAGAACAATTCA  R:CCCTACTGCTAACCACTTTTT | (GTT)_3_(GA)_3_ | 58 | 410 | KJ470844 |
| 50 | LcSSR650 | F:AAAGAAATTCTACCATCCCAAC  R:AGACGTGAGGGGTGCTAATA | (GAA)_4_...(GAA)_3_ | 57 | 299 | KJ470845 |
| 51 | LcSSR651 | F:TGACGATGCTGAAAAGATGG  R:TCAGAGCGAACAATCAATAACA | (GAA)_4_...(GAA)_4_ | 59 | 391 | KJ470846 |
| 52 | LcSSR652 | F:GTTGAATTTGTGGCTAAGTTGA  R:AGCACAAGGCTCCAACTTTT | (GTT)3 | 58 | 195 | KJ470847 |
| 53 | LcSSR653 | F:TTTCAGCCATGTCACTTTTGA  R:ACCTCCTTTGTGTAGCCAGA | (CTT)_5_ | 59 | 373 | KJ470848 |
| 54 | LcSSR654 | F:ACATGCAGTGAGTCAGCACA  R:CAAAGATTGCTTCAAAAAGCTG | (CT)_6_(GAA)_4_ | 59 | 392 | KJ470849 |
| 55 | LcSSR655 | F:CGTATGAGCCGTATTTGCAG  R:AAGTCTCTGTGGTAAGGGCTTG | (GA)_5_ | 59 | 381 | KJ470850 |
| 56 | LcSSR656 | F:GGTTGAGTCTGCTCATTGGA  R:GCTTTGTTGATTCCCCACAT | (CA)_4_ | 59 | 370 | KJ470851 |
| 57 | LcSSR657 | F:GCACATGGAGAGAATTGGTG  R:CAGGAAACAGCTATGACCATGA | (CAA)_3_ | 60 | 494 | KJ470852 |
| 58 | LcSSR658 | F:GATATCTATGGTGTCATTCT  R:CTTTTCCGTGTTTTGTTC | (CTT)_6_ | 50 | 293 | KJ470853 |
| 59 | LcSSR659 | F:CTTGTTTTGTTACTCCGTGTTC  R:CTTGATTGACTACGCCTCTAAG | (ATT)_4_ | 56 | 349 | KJ470854 |
| 60 | LcSSR660 | F:ACAACATCAAGGCGACATCC  R:CGCATAAAGACTCAACATCACA | (GAA)_3_ | 60 | 360 | KJ470855 |
| 61 | LcSSR661 | F:AAATATGTCGGTGACTGAA  R:CATGCATTACTCTTATGTCC | (GAA)_3_ | 50 | 392 | KJ470856 |
| 62 | LcSSR662 | F:TGTTCACCATCTTCCTCAACA  R:AGCAAGACAATGAATCCTCA | (GAA)_7_ | 59 | 452 | KJ470857 |
| 63 | LcSSR663 | F:CCTGGTTAGGTGATTCCCTTT  R:TTCCAACCAATGATAATTCTGC | (GT)_4_(ATT)_3_ | 59 | 488 | KJ470858 |
| 64 | LcSSR664 | F:TGACAAACGATGAACTTTTCG  R:CCAATTCACCATCATTATACGA | (GTT)_4_...(GTT)_4_ | 58 | 359 | KJ470859 |
| 65 | LcSSR665 | F:TTCATCCTTGTTGCCATTGA  R:ACAAGAGAAAAGGGGGTTGC | (CAA)_3_ | 60 | 389 | KJ470860 |
| 66 | LcSSR666 | F:AAACTTTTATTCCGCTGCTT  R:ATTGCCTGCATAGCATCAGC | (GTT)_5_ | 60 | 385 | KJ470861 |
| 67 | LcSSR667 | F:TGGACAGATGGTTGTCGAAC  R:CATCAGGGAAAACTTCAGCA | (GAA)_4_ | 59 | 374 | KJ470862 |
| 68 | LcSSR668 | F:AGGGACGGCTTCTTTTTGGA  R:TTCCATCCCTATGGAACCTT | (CAA)_3_ | 58 | 299 | KJ470863 |
| 69 | LcSSR669 | F:CAACCTGTGCAGGCTCTTTT  R:GATGAAAATCCAGGGAGCAA | (GAA)_3_... (GAA)_3_ | 60 | 330 | KJ470864 |
| 70 | LcSSR670 | F:ACTATAGAGCCACACACAAT  R:TCCAAGTCAAAATATGGTAG | (CTT)_4_ | 50 | 391 | KJ470865 |
| 71 | LcSSR671 | F:TCTCTGAGGACGATCAAACTGA  R:TATGATGCCAACGCAAAATG | (CTT)_3_ | 60 | 399 | KJ470866 |
| 72 | LcSSR672 | F:TGTCTCGTGAAGTAATCAAATG  R:GTGGAATTGGAGCGGATAAC | (GAT)_3_(TA)_3_ | 59 | 476 | KJ470867 |
| 73 | LcSSR673 | F:ATATTAGCAAGTGGACAAGA  R:AAAGCTTTATCACAACAGTG | (CT)_7_...(GAA)_5_ | 50 | 399 | KJ470868 |
| 74 | LcSSR674 | F:CATAAGAACGCTCATGCTCCT  R:CAAATGAAGATGTTGCCATTGT | (CTT)_3_...(CTT)_3_... (TA)_5_ | 59 | 421 | KJ470869 |
| 75 | LcSSR675 | F:AAACGCTGGGGCAACATA  R:ATTTCTGGCACAGGCATCTC | (GAA)_3_ | 60 | 297 | KJ470870 |
| 76 | LcSSR676 | F:TTTGAGAGGAGTTTTGTGATCG  R:ATCCTCCGGAAACACATCCT | (GAT)_3_ | 60 | 300 | KJ470871 |
| 77 | LcSSR677 | F:GCTAGTCACGAACATTCAGTTT  R:TGGGTCATCGTGCAAGTTT | (GAA)_4_..(GAA)_3_...(GAA)_3_ | 60 | 300 | KJ470872 |
| 78 | LcSSR678 | F:ACCTAAAGGACACGCCAGAA  R:CCTGCTTCACCATCCAATCT | (GAA)_3_... (GAA)_3_ | 60 | 366 | KJ470873 |
| 79 | LcSSR679 | F:TCTGAGGACGGTCAAACTGA  R:AAAAGCCATTGATTTTGCTC | (CTT)_3_ | 59 | 296 | KJ470874 |
| 80 | LcSSR680 | F:TGTCAGCACACGATTGGACT  R:CAAGAGTTGCTTCAAAAAGCTG | (CT)_7_ | 60 | 360 | KJ470875 |
| 81 | LcSSR681 | F:AATGCGCCTGGGGTATTTAT  R:AGTCAACGGTAATGCCAATTT | (GAA)_3_ | 60 | 384 | KJ470876 |
| 82 | LcSSR682 | F:AACCCTCACTCACAAAGGAA  R:GCCCAAGATATTTTGAAAGTGC | (GA)_7_ | 59 | 332 | KJ470877 |
| 83 | LcSSR683 | F:TCTCACACTCTCGATCTCCTTG  R:TTCATCTCTGGGCTCGTTCT | (GAA)_3_... (GAA)_4_ | 59 | 369 | KJ470878 |
| 84 | LcSSR684 | F:GCCTAGGCCTAATCTATGTCTT  R:TCAAATTCATTGCTCTTCCTCT | (CAA)_3_...(GAA)_4_.. (GAA)_4_ | 58 | 400 | KJ470879 |
| 85 | LcSSR685 | F:AGTTGTCCTTTGTCAATCTTTG  R:GCAAAATCCCTTACAACTTGC | (GAA)_4_ | 58 | 296 | KJ470880 |
| 86 | LcSSR686 | F:GGATGTCACATTTCAGTCTTCA  R:TGTGAAATCTCACCCCTTCTG | (CAA)_4_ | 60 | 372 | KJ470881 |
| 87 | LcSSR687 | F:ATAAATAGAGTCGCCACC  R:CTCCAGTTACCATAGTTCAG | (ATT)_3_ | 59 | 364 | KJ470882 |
